# Enhancement of drought tolerance in rice by silencing of the *OsSYT-5* gene

**DOI:** 10.1371/journal.pone.0258171

**Published:** 2021-10-22

**Authors:** Sudha Shanmugam, Virginia Ann Boyett, Mariya Khodakovskaya

**Affiliations:** 1 Department of Biology, University of Arkansas at Little Rock, Little Rock, AR, United States of America; 2 University of Arkansas Rice Research & Extension Center, Stuttgart, AR, United States of America; National Taiwan University, TAIWAN

## Abstract

Improvement of drought tolerance of crops is a great challenge in conditions of increasing climate change. This report describes that the silencing of the synaptotagmin-5 (*OsSYT-5*) gene encoding the rice Ca^2+^ sensing protein with a C2 domain led to a significant improvement of rice tolerance to water deficit stress. Transgenic lines with suppressed expression of the *OsSYT-5* gene exhibited an enhanced photosynthetic rate but reduced stomatal conductance and transpiration during water deficit stress. The abscisic acid (ABA) content under both normal and drought conditions was elevated in the leaves of the transgenic rice as compared to the wild type. The silencing of the *OsSYT-5* gene affected the expression of several genes associated with ABA-related stress signaling in the transgenic rice plants. In the water deficit experiment, the transgenic lines with a silenced *OsSYT-5* gene exhibited symptoms of drought stress seven days later than the wild type. Transgenic lines with suppressed *OsSYT-5* gene expression exhibited higher pollen viability and produced more grains compared to the wild type at both normal and drought stress conditions.

## Introduction

Plants are complex organisms, and the natural environment for plants can contain a set of both abiotic and biotic stresses. Global warming leads to the concurrence of several abiotic and biotic stresses, thus affecting agricultural productivity [1]. Abiotic stresses have a major impact on crop productivity worldwide, reducing yields in a range of crop plants [2]. Drought, heat, cold, salt, and toxic metal stresses are interconnected to osmotic stress, resulting in the disruption of ion distribution and homeostasis in the cell [2]. This disruption is mainly due to changes in the expression patterns of a group of genes which ultimately leads to responses that affect growth rates and productivity.

Plant tolerance to abiotic stress can be enhanced by various strategies including classical breeding [3] and genetic engineering [4]. The conventional breeding techniques have contributed significantly to the creation of stress-tolerant crops with high yields, however, the pace to develop new cultivars has been relatively slow and the limitation of fertility barriers allowed only the same or closely related species for hybridization [5]. The genetic engineering approach has solved these problems by producing stress-tolerant crops more efficiently and rapidly than selective breeding [6]. Moreover, genetic engineering extends the sources of genetic information, even outside the kingdoms, which could not be done by conventional breeding methods [7].

Genetic manipulation of plant signaling pathways is one of the most promising approaches for the enhancement of plant stress tolerance beyond the innate level. Understanding plant signaling pathways will allow the exploration of the specific network of genes that can be subjected to genetic manipulation in the production of abiotic stress-tolerant transgenic crops [8]. Mitogen-Activated Protein Kinase (MAPK) cascade, ABA signaling, and calcium-dependent signal transduction are the most important parts of plant stress signaling that can be modified by a genetic approach [9].

The importance of calcium (Ca^2+^) ions in calcium-dependent stress signaling pathways triggered extensive studies on the functional characterization of new Ca^2+^ sensing proteins *in planta*. Up to date over 250 Ca^2+^ sensor proteins are known in *Arabidopsis* and such proteins can be combined in three major groups: calcineurin-B-like proteins (CBLs), calmodulin (CaM)/calmodulin-like proteins (CMLs), and calcium-dependent protein kinases (CPKs), including Ca^2+^ and CaM protein kinase (CCaMK) [10]. Recently, CBL-interacting protein kinases (CIPKs) were recognized as a novel family of Ca^2+^ sensor proteins [11].

Interactions of Ca^2+^ to Ca^2+^-sensing proteins will lead to conformation changes that may result in their association with other target proteins or direct activation of the kinase activity [10, 12]. Calcium-sensing proteins may contain Ca^2+^ binding domains called CaLB (Calcium-dependent lipid-binding domain) or otherwise called C2 domains [13]. These C2 domains bind with the lipid in the cell membranes in the presence of Ca^2+^ [14]. The C2 domain is typically comprised of two, four-stranded beta-sheets creating three loops at the top of the domain and four at the bottom. Five conserved aspartate residues and one serine in upper loops 1 and 3 are involved in the binding of three Ca^2+^ ions necessary for phospholipid binding [15].

Calcium plays two important roles in the membrane targeting of the C2 domains. The first role is to provide a connection between the C2 domain and the phospholipid. The second role is to induce inter- or intra- conformational changes in the C2 domain, which in turn stimulates membrane-protein interactions [16]. It was documented that C2 domains not only play an important role in signal transduction but also in vesicle trafficking and other cellular processes [17].

Several functionally characterized C2 domain proteins are playing important roles in plant responses to abiotic and biotic stresses [17]. For example, Yang et al. showed that the C2 domain protein BAP1 can negatively regulate defense responses in *Arabidopsis* [18]. Since a majority of the C2 domain-containing proteins in plants are not functionally characterized, the discovery and characterization of such proteins can lead to the identification of new candidate genes that can be subjected to future genetic manipulations with the goal of enhancement of stress tolerance in crops.

Recently, we found that the *Arabidopsis thaliana* calcium-dependent lipid-binding protein (AtCLB) can negatively regulate the abiotic stress response in *Arabidopsis thaliana*. The loss of AtCLB function resulted in the improvement of salt and drought tolerance in T-DNA knockout mutant lines [58]. The National Center for Biotechnology Information (NCBI) Basic Local Alignment Search Tool (BLAST) demonstrated that the C2 domain of AtCLB has homology to C2 domain sequences from *Solanum lycopersicum*, *Ricinus communis*, *Sorghum bicolor*, *Populus trichocarpa*, *Vitus vinifera*, and *Oryza sativa* [58].

In *Oryza sativa* (rice), the C2 domain with strong homology to the C2 domain of AtCLB is present in synaptotagmin-5 protein (OsSYT-5). Synaptotagmins are one group of C2 domain-containing proteins that were well described in animals due to their role in neurotransmitter release [19], but poorly characterized in plants. Functional analysis of some synaptotagmins was performed using only *Arabidopsis* as a model plant. Synaptotagmin 1 (SYT1) is the first plant synaptotagmin to be characterized to date [20]. Schapire et al. proved that membrane trafficking mediated by synaptotagmin 1 (SYT1) is critical for plasma membrane (PM) integrity in plants [20]. Yamazaki et al. showed that in *Arabidopsis*, synaptotagmin 1 is required for the repair of stress-induced lesions in the PM during freezing and osmotic stresses [21].

The presence of calcium-binding domains in the synaptotagmins along with its PM localization suggests that the PM repair mechanism involves a calcium-mediated fusion of vesicles to the PM [20]. During freezing stress, the PM is ruptured due to the formation of ice crystals within it. This causes the influx of Ca^2+^ ions from the extracellular space into the cytoplasm through the damaged sites [21]. These Ca^2+^ ions bind with the C2A and C2B domains of the synaptotagmins, leading to their activation. Once activated, the synaptotagmins trigger vesicle-plasma membrane fusion leading to the resealing of the PM, thus decreasing the freezing injury and making the plant more tolerant to cold stress [21].

Lately, another research group reported that an *Arabidopsis* synaptotagmin localizes to the endosomes and regulates endosome recycling and movement protein (MP) -mediated trafficking of plant virus genomes through plasmodesmata [22]. Interestingly, *Arabidopsis* synaptotagmin 1 negatively enhances the disease resistance to *Arabidopsis*-adapted *Golovinomyces orontii* fungus, by regulating the exo/endocytosis at the PM [23]. As a result, *Syt1* mutants were more resistant to the fungus than wild-type plants [23].

There appear to be no reports on the possible functions of synaptotagmin 5 (SYT-5) in plants. This study has two aims. First, taking into consideration the strong homology between rice OsSYT-5 protein and the previously characterized AtCLB protein from *Arabidopsis* [58], this research is attempting to clarify the possible functions of *OsSYT-5* in plants using a reverse genetics approach that silenced *OsSYT-5* in rice. Second, this research assesses the biotechnological potential of manipulations with *OsSYT-5* by clarification of the effects of the suppression of *OsSYT-5* on rice phenotype and in the response of the plants to water deficit stress.

## Materials and methods

### Vector construction and transgenic rice lines development

Using a Gateway cloning strategy, the silencing construct was prepared. Frozen leaves of rice cultivar *LaGrue* were used for the isolation of total RNA using the RNeasy Plant Mini Kit (Qiagen Inc., Germantown, MD). Synthesis of complementary DNA (cDNA) was performed according to the kit protocol using the SuperScript III First-Strand Synthesis System Kit (Invitrogen) with oligo (dT) primers. The cloning insert, a 276 base pair (bp) fragment from position 1445 to 1720 of the *OsSYT-5* gene (*Os07g0409100* sequence) was synthesized by PCR using *OsSYT-5* specific primers and the established cDNA. The sequences of the primer pair selected were 5’-CACCGTTGGACTTGTGGGCACT-3’ (forward primer) and 5’-TGCGATGTCCATTGCAATCACTGTA-3’ (reverse primer). The insert included 32 bases downstream of the stop codon. The original first four bases of TGGT on the 5’ end of the forward primer were replaced with CACC to facilitate directional Gateway Cloning. The PCR was performed using high fidelity Platinum *Pfx* DNA Polymerase (Invitrogen) in 35 cycles of a traditional 3-step PCR protocol with an annealing temperature of 59°C and an extension temperature of 68°C. The PCR product was resolved on a 1% agarose gel in 1 X TBE, stained with ethidium bromide. The gel was visualized and recorded using a Gel Doc XR Gel Imaging System (Bio-Rad Life Science Research, Hercules, CA). The *pENTR_OsSYT*-5 Entry clone was prepared with the Gateway entry vector pENTR /D-TOPO (Invitrogen, Inc.) and the 276 bp *OsSYT-*Pfx product as the insert DNA. Transformation of One Shot TOP10 Chemically Competent *E*. *coli* (Invitrogen) resulted in 27 colonies on the kanamycin selective LB agar medium. All colonies were confirmed to have the insert through PCR using the *OsSYT-5* specific primers (S1B Fig). To confirm the sequence and direction of the *OsSYT-5* insert, a sample of the purified plasmid of PCR-confirmed clone *pENTR_OsSYT-5* was sequenced at the DNA Sequencing Core Facility at the University of Arkansas for Medical Sciences. The cloning region was sequenced with M13 primers in both directions, yielding two distinct sequences. The sequences were then compared with the published *OsSYT*-5 sequence using an online alignment tool, *EMBOSS Water Pairwise Sequence Alignment*. The length of alignment was 276 bp with both identity and similarity at 100% (S1A Fig). The verified pENTR_*OsSYT*-5 plasmid was used in a Gateway LR Clonase Enzyme (Invitrogen) reaction to transfer the 276 bp *OsSYT-5* gene insert into the destination vector pANDA [24] (S2 Fig), thus creating the silencing construct. The pANDA vector was kindly provided by Dr. K. Shimamoto. After overnight incubation at room temperature, the reaction was stopped with the addition of Proteinase K and then used to transform One Shot TOP10 competent *E*. *coli* using the standard 42°C heat shock protocol. Hygromycin B- resistant isolated colonies were tested for the presence of the *OsSYT-5* insert using PCR with the *OsSYT-5* insert specific primers. Confirmed colonies were selected for growth in 100 ml LB medium (kanamycin/hygromycin selective) and subsequent plasmid isolation. Liquid cultures were incubated shaking at room temperature. Plasmid purifications were accomplished using the QIAfilter Plasmid Midi kit (Qiagen, Inc.) according to kit instructions, and the purified pANDA_*OsSYT-5* DNA stocks were confirmed to have the *OsSYT-5* fragment through PCR analysis and double restriction digest using *KpnI* and *SacI* restriction endonucleases. Digestion and PCR products were resolved on a 1% agarose gel in 1 X TBE, stained with ethidium bromide. The gel was visualized and recorded using a Gel Doc XR Gel Imaging System (Bio-Rad). The confirmed silencing construct pANDA-*OsSYT-5* was submitted to the Iowa State University Plant Transformation Facility for the generation of the transgenic *Nipponbare* rice lines.

### Analysis of expression of the *OsSYT-5* gene in established transgenic rice lines

Plants of 14 established *Nipponbare* rice lines transformed with the pANDA-*OsSYT-5* silencing construct were analyzed for expression of the *OsSYT-5* gene. Total RNA from the leaves was isolated using the RNeasy Plant Mini Kit (Qiagen Inc.). Residual DNA was removed by on-column DNA digestion using the RNase-free DNase kit (Qiagen Inc.). Synthesis of cDNA was carried out using SuperScript III First-Strand Synthesis System Kit (Invitrogen) with dT16-oligonucleotide primers according to the manufacturer’s protocol. Following synthesis, each sample was diluted 10 times and 4 μl of the diluted cDNA sample was used for the quantification of *OsSYT-5* gene expression using real-time quantitative RT-PCR analysis (qRT-PCR). For the reaction, SYBR Green PCR master mix (Applied Biosystems, Carlsbad, CA, USA) was mixed with the cDNA and primers and transferred in an iCycler iQ Multi-Color Real-Time PCR detection system (Bio-Rad, Hercules, CA, USA) in a total reaction volume of 25μl. The following primers were used:

5’-CACCGTTGGACTTGTGGGCACT-3’ (F);
5’-TGCGATGTCCATTGCAATCACTGTA -3^’^ (R) to amplify *OsSYT-5* and
5’-AGGCCGCGGAAGTTTGAGGC-3’ (F); 5’-ATCAGTGTAGCGCGCGTGGG -3’ (R)

to amplify 18S, a housekeeping gene used as a gene control. Three independent biological replicates were used in the analysis. The real-time PCR data were generated and analyzed to obtain the relative mRNA expression. The comparative delta delta Ct method was used for the analysis. The Ct values obtained from the samples were normalized to an 18S housekeeping gene and then compared. First, the difference between the Ct values (delta Ct) of the gene of interest (*OsSYT-5*) and the house keeping gene was calculated for each sample (wild and transgenic). Then the difference in the Ct values between the experimental (transgenic) and control (wild) samples (delta delta Ct) was calculated. Finally, the fold change in the expression of the gene of interest between the wild type and the transgenic lines was calculated as 2^ (-delta delta Ct). After the statistical analysis, data were plotted into a graph that shows the relative transcript abundance of the transgenic lines compared to the WT line.

### Water deficit experiments involving the transgenic rice lines with silenced *OsSYT-5* gene

For the drought tests, 3-week-old rice plants were transferred to the Sungro professional growing mix (1-gallon bucket) and grown under standard growth conditions (14-h-light/10-h-dark cycle at 28°C). The plants were subjected to drought conditions by withholding water for 63 days. For each line, a minimum of 10 plants was included in the experiment. During the drought stress experiment, the volumetric water content (VWC) of the soil was measured at 0, 5, 7, and 9 weeks using the ProCheck decagon device (Decagon Devices, Inc., USA). The volumetric water content is the ratio of the volume of water to the unit volume of soil expressed as a ratio or percentage. To evaluate the water loss rate, the topmost fully expanded leaves were detached from each plant and weighed at different time intervals at room temperature. The proportion of fresh weight loss was calculated based on the initial weight of the leaf. The relative water content (RWC) of the detached leaves was measured by the whole leaf method [25]. Six leaves were cut from each 6-week-old plant grown in a greenhouse. The top and bottom portions were cut off and the middle 5cm section remaining was used for the experiment. The fresh weight was measured immediately after cutting. The leaves were dipped in distilled water and kept in a refrigerator at 4°C for 24 hours to reach full turgor. The turgid weight and, later, the dry weight were measured after drying the leaves at 70°C for 24 hours. The RWC was calculated as a percentage by the formula: RWC (%) = [(fresh weight-dry weight)/(turgid weight-dry weight)] X 100. The data were analyzed statistically by one-way ANOVA (Analysis of Variance) with posthoc Tukey HSD (Honestly significant difference) using SAS software.

### ABA analysis in the leaves of WT and transgenic rice lines

Abscisic acid (ABA) measurements were conducted using the Phytodetek ABA test kit (Agdia Inc.). Measurements were performed in 3-month-old wild-type and transgenic lines according to [26]. Briefly, 1–2 g of the leaves of 3-month-old plants of wild type and transgenic lines grown in greenhouse conditions were frozen in liquid nitrogen, powdered, and mixed in 5ml of 80% methanol. The samples are stored at 4°C for 4 hours and then centrifuged at 4°C for 15 min. at 4000 rpm. The supernatant was collected and used for the measurements. The ABA quantification was repeated in leaves of the wild type and transgenic lines following a drought treatment (withholding water for 30 days) as described above.

### Analysis of the expression of selected genes associated with ABA signaling in rice wild type and transgenic lines with silenced *OsSYT-5* gene grown under normal and water deficit conditions

The expression of 13 selected rice genes (*OsZEP*, *OsWRKY-45*, *OsHsfA*, *OsSKC-1*, *OsAKT-1*, *OsCAX*, *OsTPC-1*, *OsGMST-1*, *OsPIP1-1*, *OsPIP1-2*, *OsHsp-70*, *OsSIK-1*, *OsCPK-2*) was analyzed using qRT-PCR in the wild type and three transgenic rice lines with the *OsSYT-5* gene silenced (16–4, 32–4, 2–1). Conditions of qRT-PCR were as described above. The primers that were used in the reaction are described in Table 1. Three independent biological replicates were used in the analysis. The real-time PCR data were generated and analyzed to obtain the relative mRNA expression.

**Table 1:** Primers used for the amplification of *OsZEP*, *OsWRKY-45*, *OsHsfA*, *OsSKC-1*, *OsAKT-1*, *OsCAX*, *OsTPC-1*, *OsGMST-1*, *OsPIP1-1*, *OsPIP1-2*, *OsHsp-70*, *OsSIK-1* and *OsCPK-21* genes.

***OsZEP-1***	Forward Primer	TCGTCTCCTCAGATGTCGGT
	Reverse Primer	TCTCCACCCATGGCTTGTTC
***OsWRKY-45***	Forward Primer	GGGAATTCGGTGGTCGTCAA
	Reverse Primer	GAAGTAGGCCTTTGGGTGCT
***OsHsfA***	Forward Primer	GCAGCTCAACACCTACGGAT
	Reverse Primer	ACGAACCATCCTGCTTTGGT
***OsSKC-1***	Forward Primer	CAAACACCAGCAAACGGGAG
	Reverse Primer	CTTGAGCCTGCCGTAGAACA
***OsAKT-1***	Forward Primer	ACTATTAGTCGAAGGCGGCG
	Reverse Primer	ATCATGGGCTCGCTGTTGAA
***OsCAX***	Forward Primer	TTGTCATCGGCGCATGTTTT
	Reverse Primer	AGCTACTCTGTCACACCAGAT
***OsTPC-1***	Forward Primer	GGTGCCTACTGGATGGAAGG
	Reverse Primer	CTCTGAAACGCCGAACTTGC
***OsGMST-1***	Forward Primer	ATGCGGTGGAAGCTTAAGTC
	Reverse Primer	TCGCCCTTACTGGTCACA
***OsPIP1-1***	Forward Primer	TGATCTTCGCGCTCGTCTAC
	Reverse Primer	GACCAGGAACACCGCAAAAC
***OsPIP1-2***	Forward Primer	CTTCCAGAAGGGCCTGTACG
	Reverse Primer	CGGAGAAGACGGTGTAGACG
***OsHsp-70***	Forward Primer	CGAGTTGGTGAAGCACTCCT
	Reverse Primer	CTCCTCAAATCGGGATCGGG
***OsSIK-1***	Forward Primer	CTGGCCCGTGGTACTTTGAT
	Reverse Primer	AGAGCTTGCATCAGGCCAAT
***OsCPK-21***	Forward Primer	TTCGCGAACAAATCGGAGGA
	Reverse Primer	ATGGCTCCCTGTTGAAGTCG

### Measurements of leaf stomatal density, photosynthetic rate, transpiration rate, and Water Use Efficiency (WUE)

To measure stomatal density, 6 to 7 leaves of the same age and from the same position were used from plants of the wild type and transgenic plants grown under similar conditions. Photosynthesis (PR), stomatal conductance (SC), and transpiration rate (TR) were measured using a portable photosynthesis system (Li-Cor LI-6400XT). Three measurements were made for each plant. Ten wild-type plants and 10 plants of each transgenic line were used for the experiments. The data were analyzed by One-way ANOVA (Analysis of Variance) with post-hoc Tukey HSD (Honestly significant difference) using SAS software. Water use efficiency (WUE) was calculated from the obtained photosynthetic and transpiration rates by using the formula: WUE = PR/TR.

### Analysis of pollen viability

To observe starch accumulation, the pollen grains from the transgenic as well as the wild-type plants were collected and stained with 1% iodine-potassium iodide solution. Then the pollen grains were directly examined under an Amscope microscope. The deeply stained and round pollen grains were counted as viable and pictures were taken.

### Phenotypical analysis of root system of the transgenic rice lines

Root morphology of the transgenic, as well as the wild-type plants, were studied following the drought condition. Root length, number of root branches, and root mass were measured and the data were analyzed statistically by one-way ANOVA (Analysis of Variance) with post-hoc Tukey HSD (Honestly significant difference) using SAS software.

#### Accession numbers

Genes from this article can be found in the National Center for Biotechnology Information (https://www.ncbi.nlm.nih.gov/) under the following accession numbers: *OsSYT-5*(Os07g0409100), *OsZEP*(AB050884), *OsWRKY-45*(AY870611), *OsHsfA*(XM_015774935), *OsSKC* (AAZ76552), *OsAKT-1*(XM_015766208), *OsCAX* (AAM03123), *OsTPC-1*(AB100696), *OsGMST-1*(AK060819), *OsPIP1-1*(XM_015769907), *OsPIP1-2*(XM_015779980), *OsHsp-70*(XM_026021101), *OsSIK-1*(GQ423058), *OsCPK-21*(EU709762).

## Results

### Suppression of *OsSYT-5* gene expression in the transgenic lines

**Fig 1:**
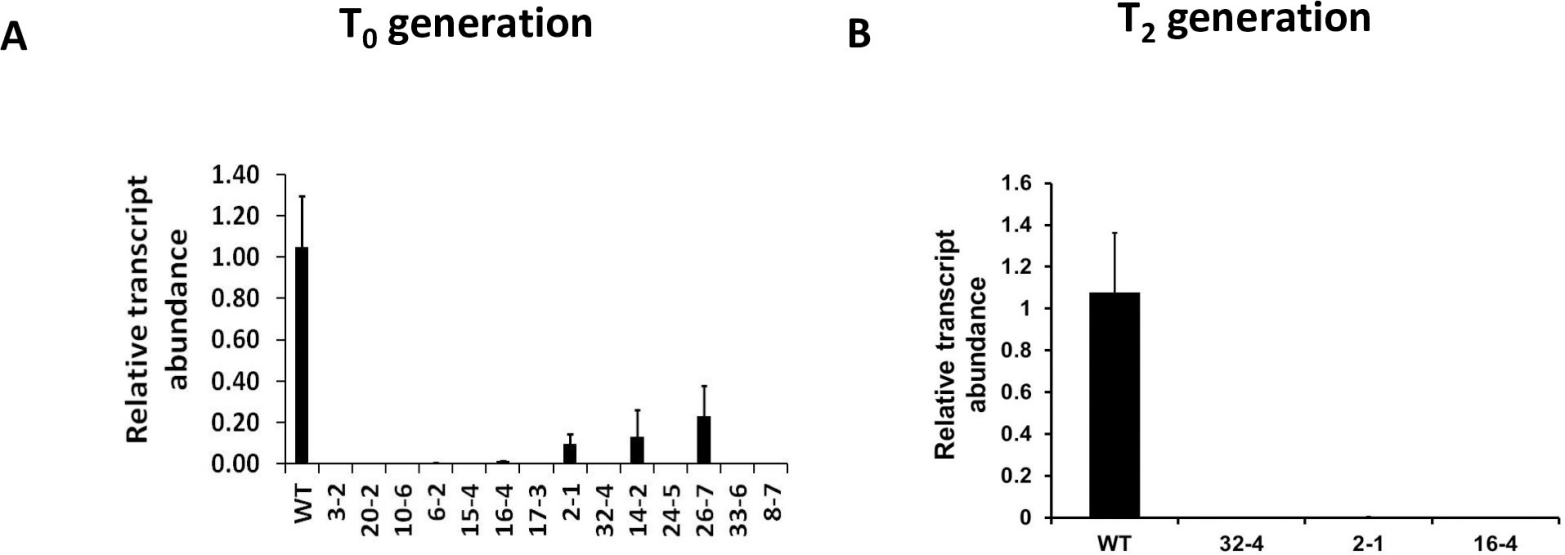
Confirmation of suppression of *OsSYT-5* gene expression in all putative transgenic lines (T_0_) (A) and three selected T_2_ rice lines (B) using real-time PCR.

A silencing construct was prepared by cloning a 276 bp fragment of the *OsSYT-5* gene (*Os07g0409100* sequence) into the silencing vector *pANDA* using the Gateway cloning strategy [27]. The established construct was provided to the Iowa State University Transformation Facility for the generation of transgenic *Nipponbare* rice lines. As a result, 14 transgenic lines carrying the *pANDA-OsSYT-5* silencing construct were generated. All generated T_0_ seedlings were heterozygous. To confirm that the natural expression of the *OsSYT-5* gene was successfully terminated in the 14 putative transgenic lines (T_0_), all the lines were analyzed by real-time PCR using primers specific to the *OsSYT-5* gene. It was found that the *OsSYT-5* expression was fully or significantly suppressed in all analyzed putative transgenic lines (T_0_) when compared to the *OsSYT-5* gene expression in the wild-type rice (Fig 1A). Three representative transgenic lines (*32–4; 16–4; 2–1*) were selected for further experiments. To prove that suppression of *OsSYT-5* gene expression is stable in generations, the expression of the transgene in both the T_1_ and T_2_ generations was also monitored. The absence of *OsSYT-5* expression in the three selected transgenic lines in the T_2_ generation is shown in Fig 1B.

### Effect of *OsSYT-5* gene suppression on the phenotype of the generated transgenic rice lines

**Fig 2:**
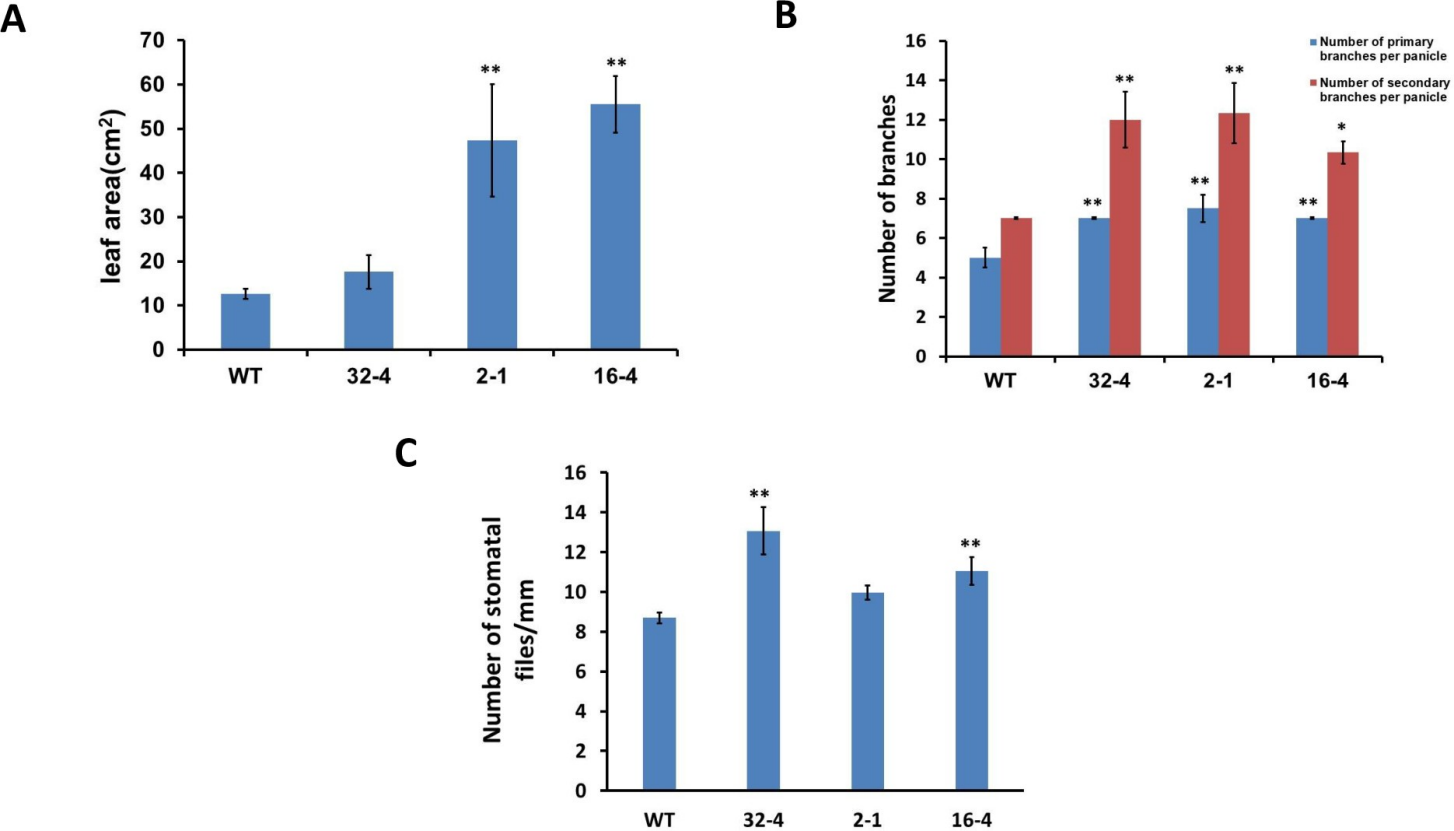
Effect of suppression of *OsSYT-5* gene on leaf area (A), number of primary and secondary branches per panicle (B), and number of stomatal files on the abaxial side of leaves (C) of 57-d-old wild type and transgenic lines. Values are mean ± SE (**P, 0.01). Thirty leaves from 10 plants of each transgenic line and wild type were used for leaf area measurement. Values are mean ± SE (*P, 0.05, **P, 0.01). Data were analyzed statistically by one-way ANOVA (Analysis of Variance) with post-hoc Tukey HSD (Honestly significant difference) using SAS software.

Phenotypical traits of generated rice lines grown in the greenhouse were assessed. The surface area of the leaves contributes to grain yield [28]. The leaf area of the three selected transgenic lines (*32–4; 2–1; 16–4*) was measured by using a portable leaf area meter Android [Biovis Leaf Av (Android version), Expert Visions Labs Pvt. Ltd, India]. The experiments proved that two of the three analyzed transgenic lines (lines *2–1* and *16–4*) exhibited a significantly larger leaf area than the wild type (Fig 2A). Additionally, the number of primary and secondary branches was significantly increased in all three tested transgenic lines (Fig 2B). It was also observed that the total number of stomatal files was increased in the transgenic lines compared to the control plants (Fig 2C). Documented effects of the suppression of the *OsSYT-5* gene expression on the size of the leaves and stomatal density may indicate that photosynthetic efficiency is different between the transgenic rice lines and the wild type. To prove this suggestion, the PR (5A) and WUE (5D) were measured in the control and transgenic lines grown in controlled environmental conditions (greenhouse).

### Changes in the root architecture in the transgenic rice lines with silenced *OsSYT-5* gene

**Fig 3:**
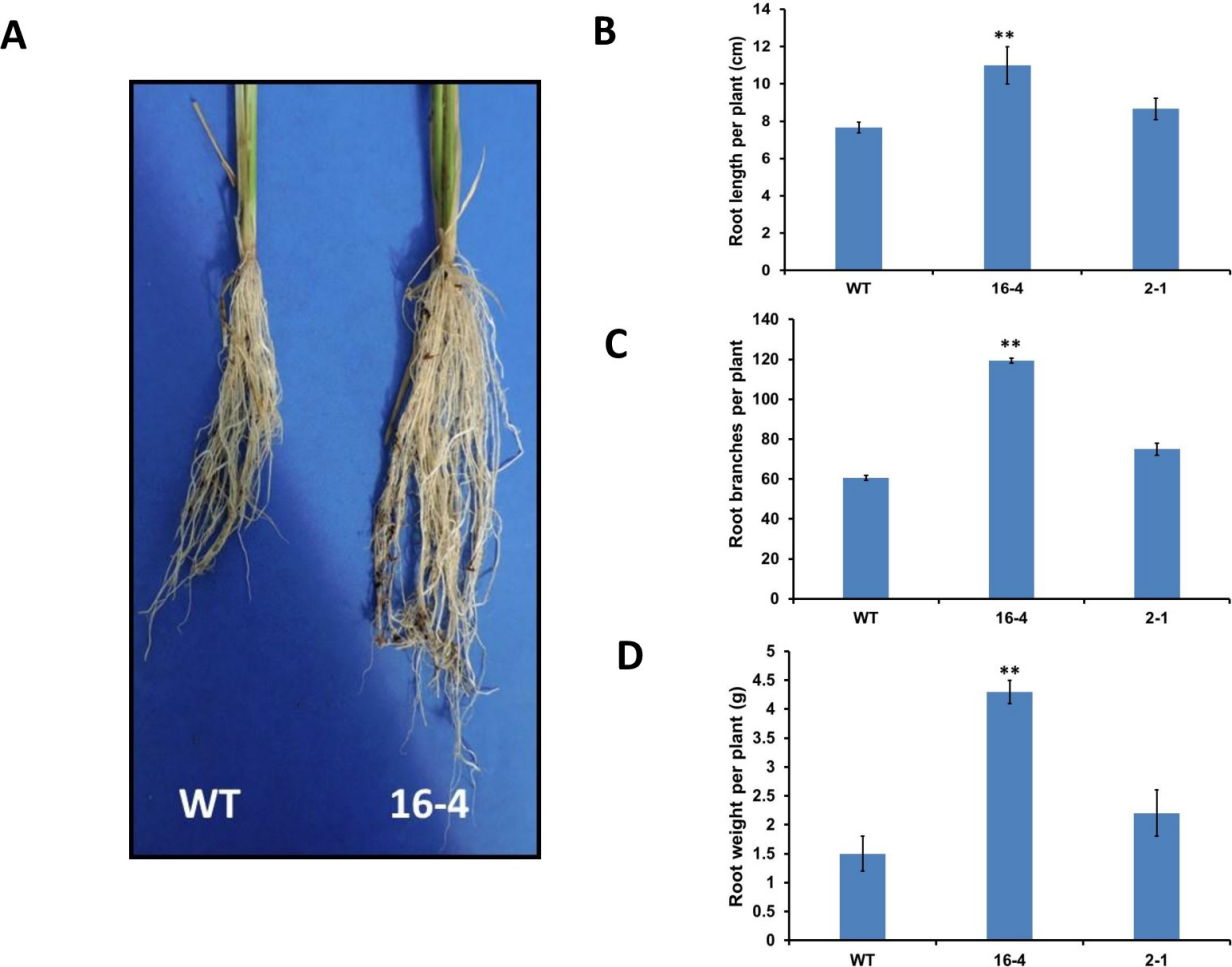
Transgenic rice lines with silenced *OsSYT-5* gene produce a deeper and thicker root system compared to the WT-type plants. The root system of 8-week-old control and transgenic plants (A). Comparison of the root length (B), the number of root branches per plant (C), and root weight per plant (D). Ten 8-week-old plants were used for each line. Values are mean ±SE (**P, 0.01). Data were analyzed statistically by one-way ANOVA (Analysis of Variance) with post-hoc Tukey HSD (Honestly significant difference) using SAS software.

Studies have proved that deeper roots increase the ability of the plant to absorb water from the soil during water-deficit stress conditions [29]. Therefore, the root morphology of the transgenic, as well as the wild-type rice, was examined. As shown in Fig 3A, the transgenic line (*16–4*) produced a larger root system compared to that of the wild type with markedly increased root length (Fig 3B) and the number of root branches (Fig 3C). The total root biomass of the transgenic line *16–4* was significantly higher than that of the control plant (Fig 3D). This observed improved root architecture may contribute to the increased tolerance of the transgenic lines to water deficit stress by increasing the soil water uptake through deeper roots.

### Effect of *OsSYT-5* gene suppression on the response of rice to water deficit stress

**Fig 4:**
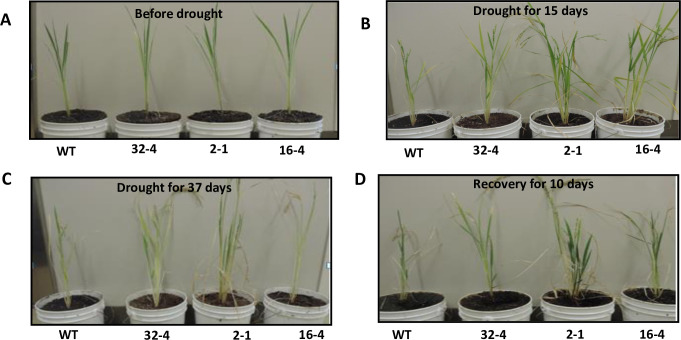
Phenotype of wild type and transgenic rice lines before water deficit experiment (A), after 15 days of water deficit stress (B), after 37 days of stress (C), and phenotype of recovered plants after 10 days of re-watering (D). 3-week old T_2_ transgenic lines and 3-week old wild type were used for the experiment.

To understand if suppression of the *OsSYT-5* gene can lead to changes in the response of rice plants on water deficit stress, drought stress experiments were performed. As a first step, 3-week-old seedlings of the established transgenic lines were tested for drought tolerance. As shown in Fig 4A, no obvious difference was observed between the control and the transgenic lines before drought stress. After 15 days of full water withholding, both the wild type and the transgenic plants were still healthy (Fig 4B). However, after 30 days of water withholding, the transgenic lines showed less leaf rolling than the wild type. At this point, the wild-type plants started to exhibit symptoms of wilting (Fig 4C). While signs of wilting were seen in the wild-type plants on the 30^**th**^ day of drought stress, the transgenic lines did not begin to show visual stress symptoms until the 37^**th**^ day of drought stress. Water was restored in 5 pots after 44 days of water-deficit stress, and in the remaining 5 pots, the VWC measurement was continued till 9 weeks. After subsequent recovery by re-watering, all tested lines recovered, but the transgenic lines recovered one week earlier than the wild type (Fig 4D). We concluded the recovery of plants on the base of the appearance of new leaves and the reversal of leaf rolling, which were noted a week early in the transgenic lines compared to the WT plants.

In the initial days of drought stress experiments, the volumetric water content of the pots containing both the wild type and the transgenic lines was almost equal (80%). The observed unusual high VWC can be explained by specific conditions of the cultivation of rice plants. Rice plants were grown in pots that were fully covered with a deep layer of water. During the drought experiment, we observed that the pots used for the cultivation of transgenic plants maintained more moisture than pots with wild-type plants during the first 9 weeks of cultivation without watering (S3 Fig).

### Photosynthetic rate and WUE are improved in *OsSYT-5* silenced transgenic plants grown under drought stress

**Fig 5:**
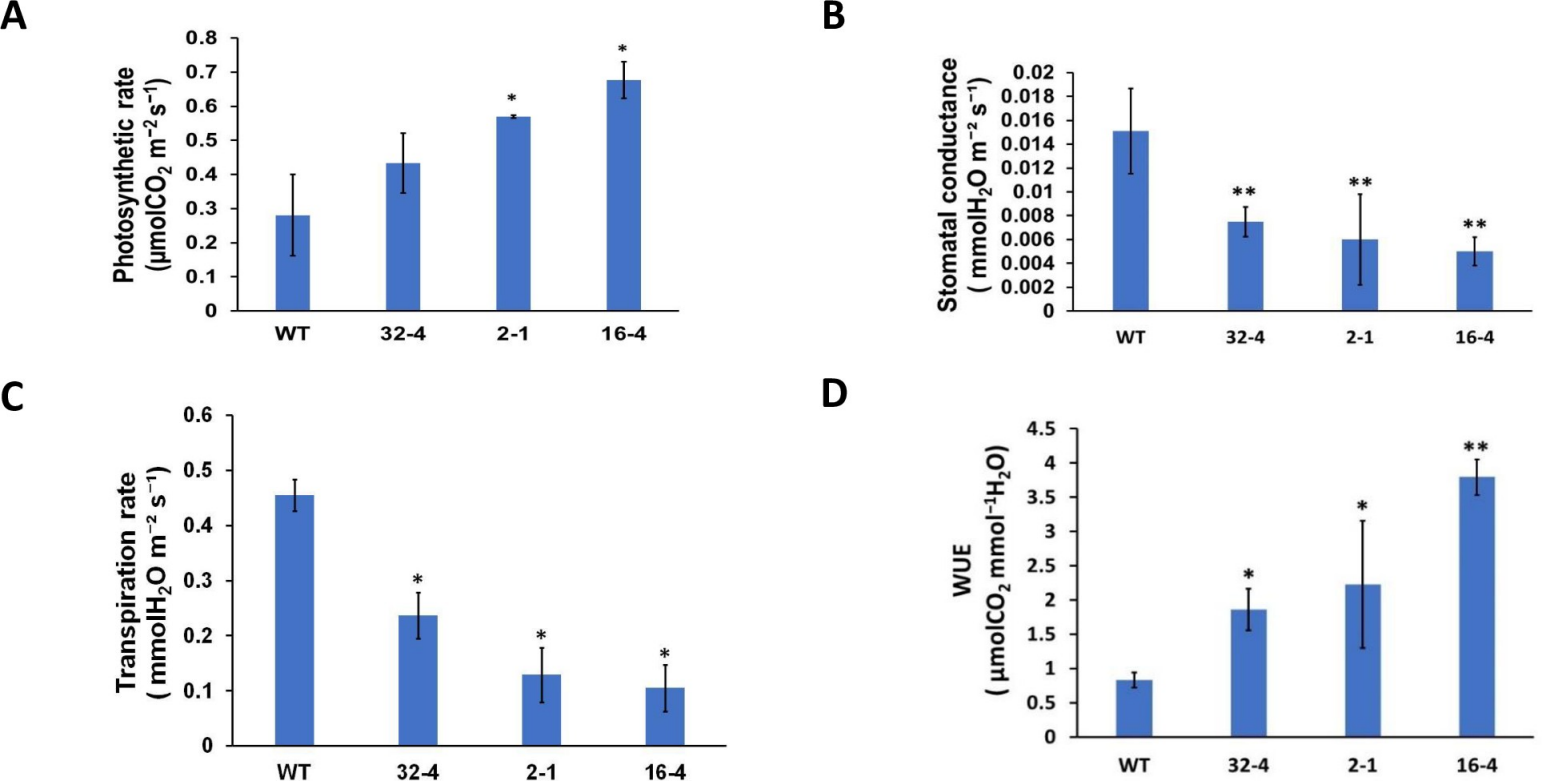
Effects of suppression of *OsSYT-5* gene on the photosynthetic rate (A), stomatal conductance (B), transpiration rate (C), and WUE (D) in the rice transgenic plants grown under water deficit stress for 22 days. Five measurements were carried out for each plant, and 10 plants were used for each line. Values are mean ± SE (*P, 0.05, **P, 0.01). Data were analyzed statistically by one-way ANOVA (Analysis of Variance) with post-hoc Tukey HSD (Honestly significant difference) using SAS software.

**Fig 6:**
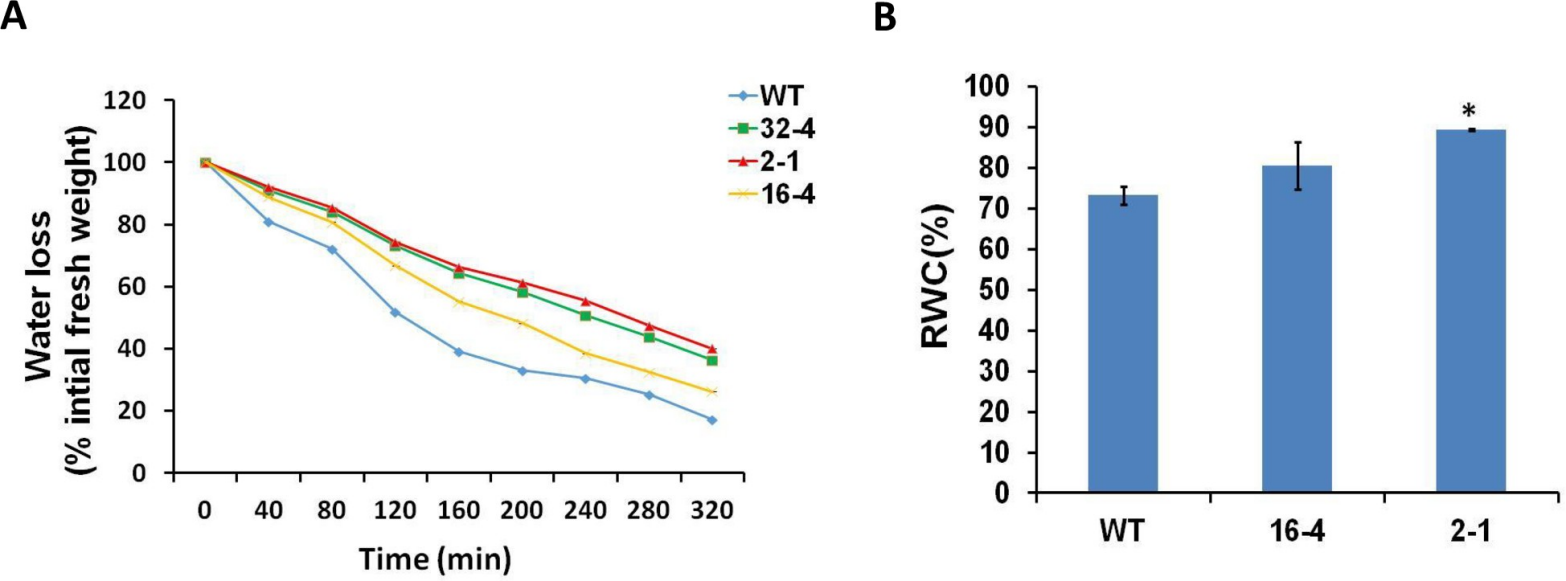
The water loss rate of detached leaves (A) and Relative Water Content (RWC) of detached leaves (B), produced by WT type and transgenic lines with silenced *OsSYT-5* gene. 51-days-old plants grown under water deficit stress for 30 days were used for water loss experiments and 90-days-old plants grown under regular watering conditions were used for RWC measurements. Each data point represents the mean of duplicate measurements. Values are mean ±SE (*P, 0.05). Data were analyzed statistically by one-way ANOVA (Analysis of Variance) with post-hoc Tukey HSD (Honestly significant difference) using SAS software.

Transgenic lines with suppressed *OsSYT-5* expression exhibited an enhanced photosynthetic rate (Fig 5A) during water deficit. At the same time, stomatal conductance and transpiration rate were reduced in all tested transgenic lines compared to the wild type (Fig 5B and 5C). Thus, it was not surprising that the WUE was improved in the transgenic lines (Fig 5D). Consistent with this result, the water loss of the detached leaves of the transgenic lines was significantly less compared to that of the wild-type plants cultivated under water deficit for 30 days (Fig 6A). The RWC of the leaves in the transgenic lines grown under regular watering was also significantly higher (an increase of 22.14%) than in the wild-type leaves (Fig 6B).

Observed significant modification of rice transpiration is crucial because it can lead to changes in the phenotypical response of transgenic lines to water deficit stress.

### Effect of *OsSYT-5* gene suppression on rice grain yield under regular and water deficit conditions of cultivation

**Fig 7:**
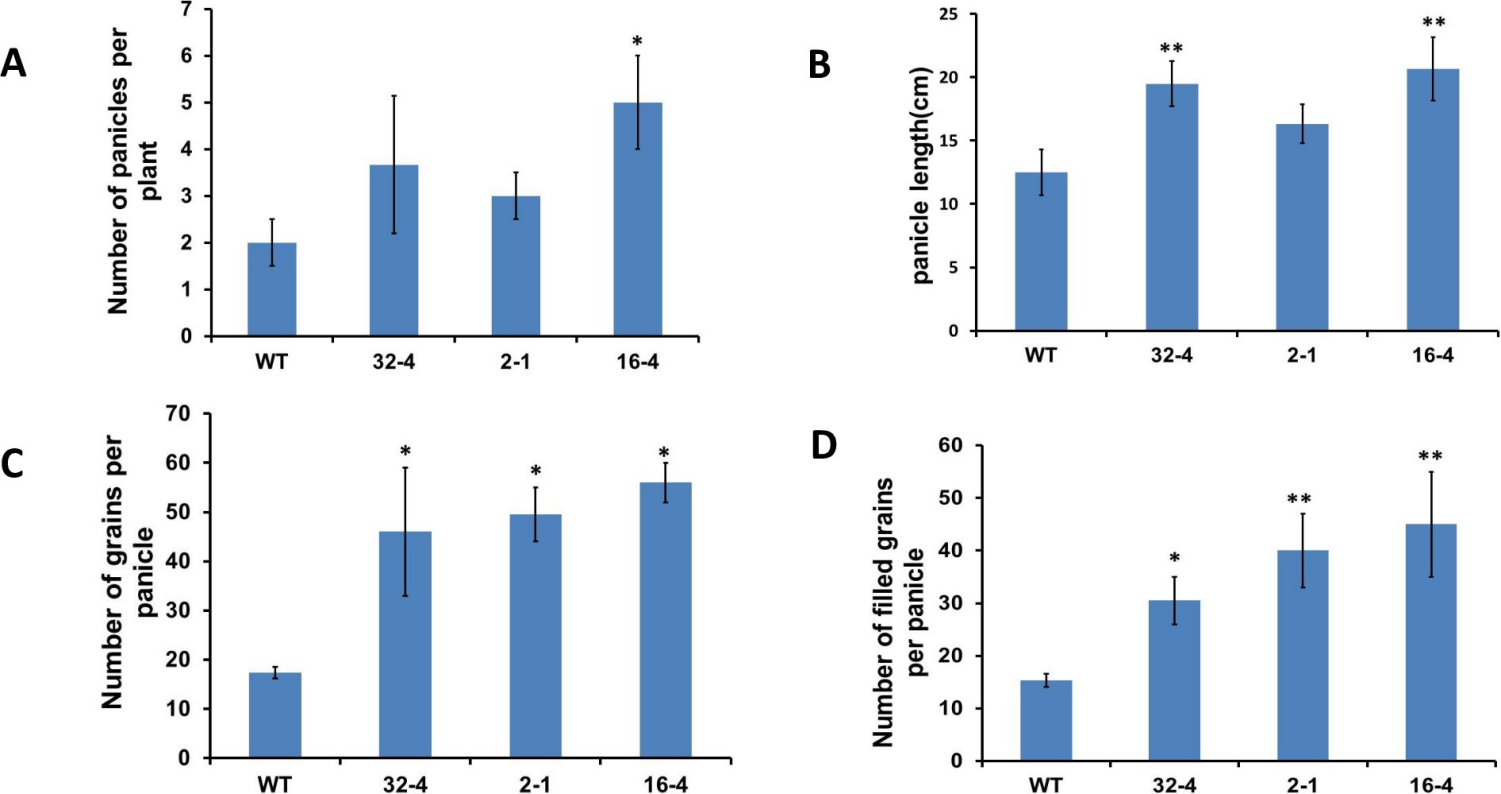
A to D, Increased panicle number (A), panicle length (B), the number of grains (filled +half-filled + unfilled) per panicle (C), and the number of filled grains per panicle (D) in the 51-day-old transgenic rice compared to that of wild-type in regular conditions in the greenhouse. Values are mean ±SE (*P, 0.05, **P, 0.01). Data were analyzed statistically by one-way ANOVA (Analysis of Variance) with post-hoc Tukey HSD (Honestly significant difference) using SAS software.

**Fig 8:**
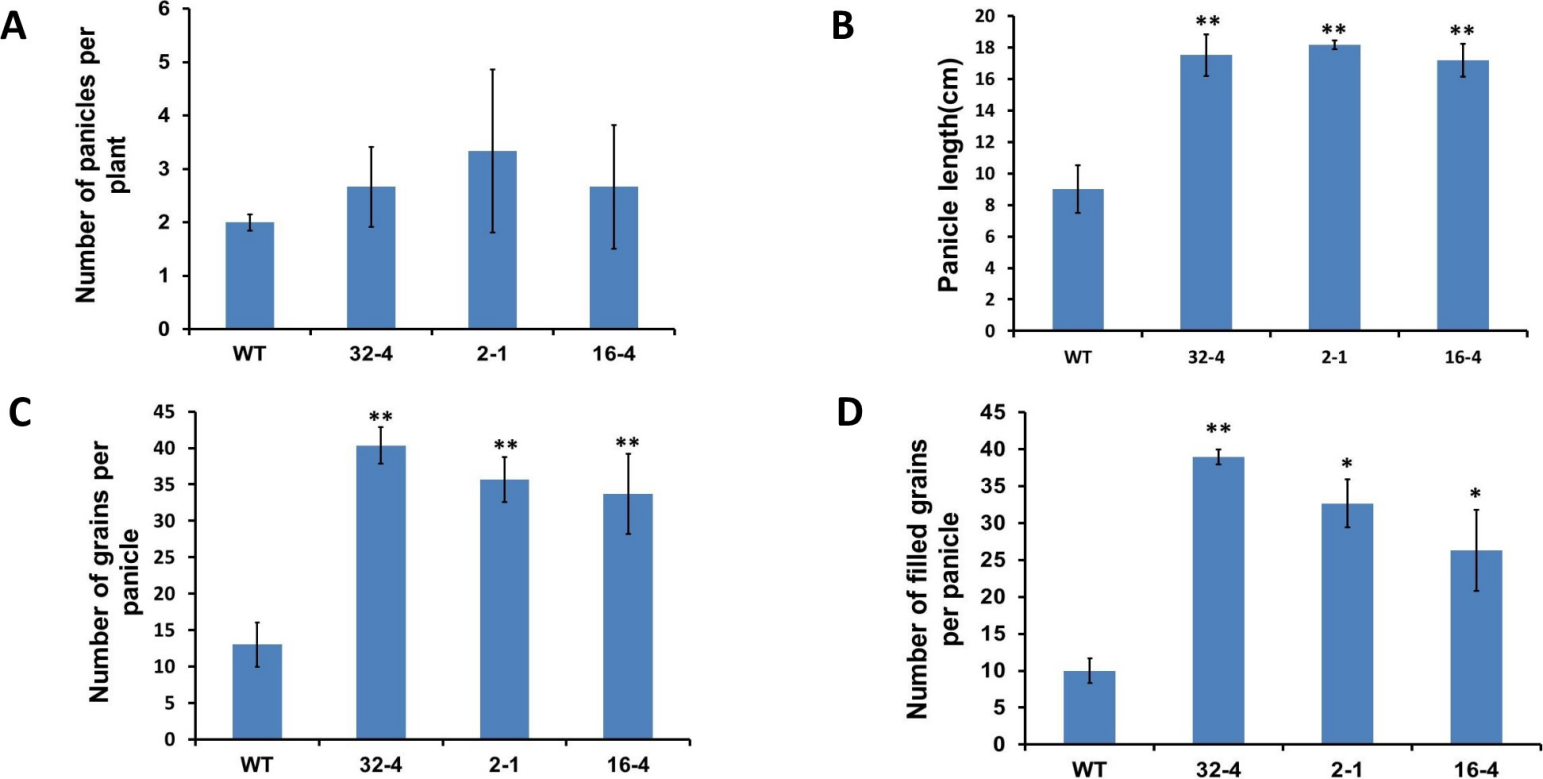
A to D, Increased panicle number (A), panicle length (B), the number of total grains (filled +half-filled + unfilled) per panicle (C), and the number of filled grains per panicle (D) in the 51-day-old transgenic rice compared to that of wild-type after 30 days of water deficit stress. Values are mean ±SE (*P, 0.05, **P, 0.01). Data were analyzed statistically by one-way ANOVA (Analysis of Variance) with post-hoc Tukey HSD (Honestly significant difference) using SAS software.

Grain yield is one of the important agronomic traits of crop plants [30] and can vary depending on different growth conditions such as climate, fertilizer used, planting period, etc., [31]. This study found that the silencing of the *OsSYT-5* gene can potentially increase grain yield and improve major yield components in rice. Thus, the number of panicles per plant (Fig 7A), length of each panicle (Fig 7B), the number of grains per panicle (Fig 7C), and the number of filled grains per panicle (Fig 7D) were higher in the transgenic lines compared to the wild-type under greenhouse conditions. The same parameters were also higher in the transgenic lines exposed to 37 days of water deficit stress (Fig 8A–8D). Correspondingly, the seed setting rate increased in the transgenic line compared to that of the wild-type. The seed setting rate is directly proportional to the number of filled grains in the plant. Since the number of filled grains was higher in the transgenic lines, it follows that the seed setting rate would also be higher.

**Fig 9:**
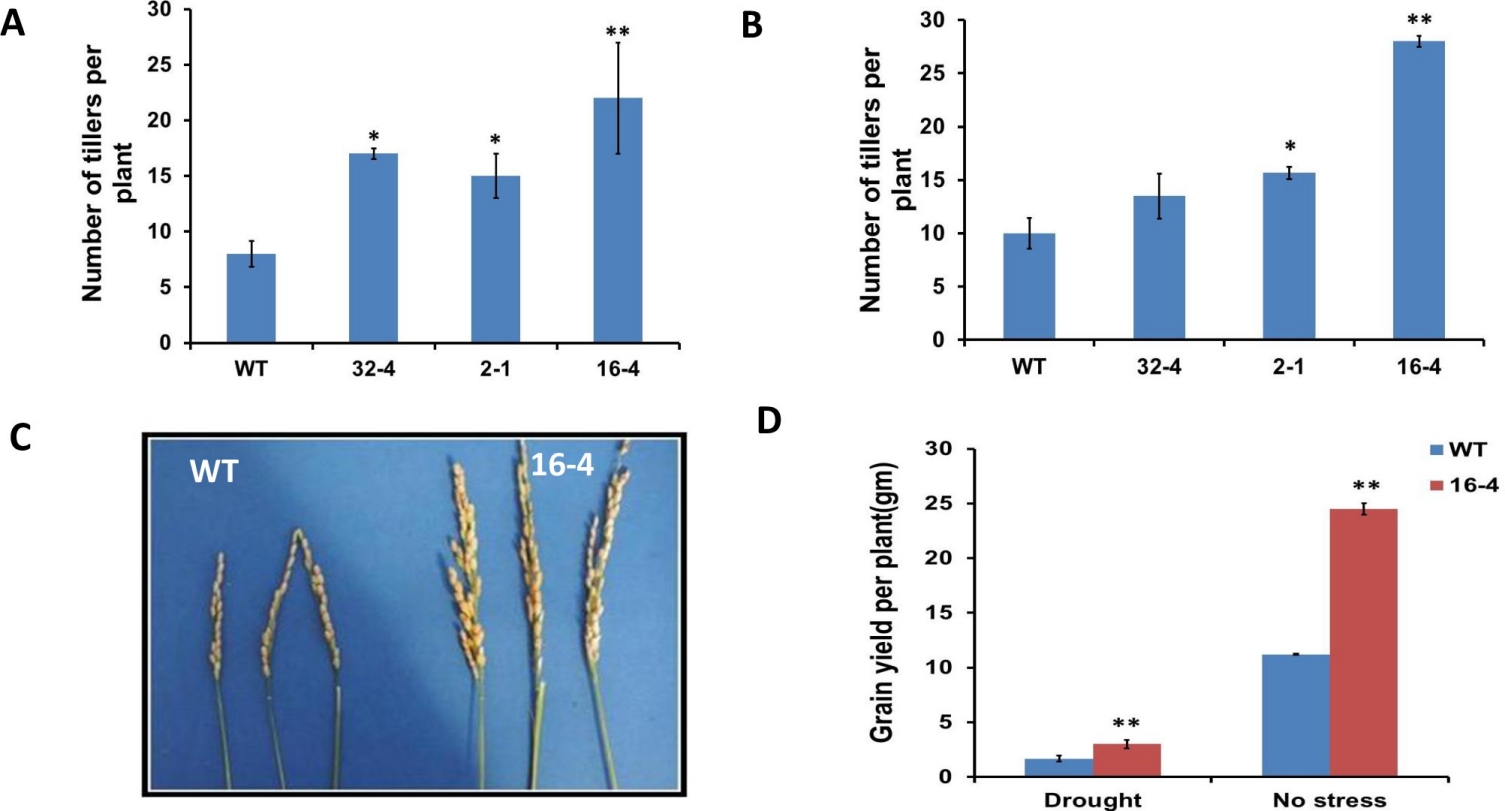
Tiller number of plants in WT type and transgenic rice lines in the seedling stage (A) and in the stage of maturity (B). The phenotype of the panicles in the transgenic rice and wild-type (C) and grain yield in the transgenic rice compared to that of the wild-type under both regular and 30 days of drought conditions (D). Ten plants were used for each line. Values are mean ±SE (*P, 0.05, **P, 0.01). Data were analyzed statistically by one-way ANOVA (Analysis of Variance) with post-hoc Tukey HSD (Honestly significant difference) using SAS software.

Tillers are the productive branches that are crucial for grain production. Thus, an increased tiller number is a major contributor to increased grain yield. The tiller number of 6-week-old wild-type and transgenic plants grown in standard greenhouse conditions was assessed (Fig 9). The results indicated that the transgenic lines 2–1, 16–4, 32–4 had significantly more tillers per plant than the wild-type plants (Fig 9A) at the 6-week stage. At maturity (15-week-old plants), the tiller number of the transgenic lines 2–1 and 16–4 was also higher than the wild-type (Fig 9B).

As shown for the representative transgenic line 16–4, the transgenic plants had larger panicles than the control (Fig 9C) and produced potentially more grains (total seed weight) at greenhouse conditions (an increase of 118.75% compared to WT) and after 37 days of water deficit stress (an increase of 87.5% compared to WT) (Fig 9D).

**Fig 10:**
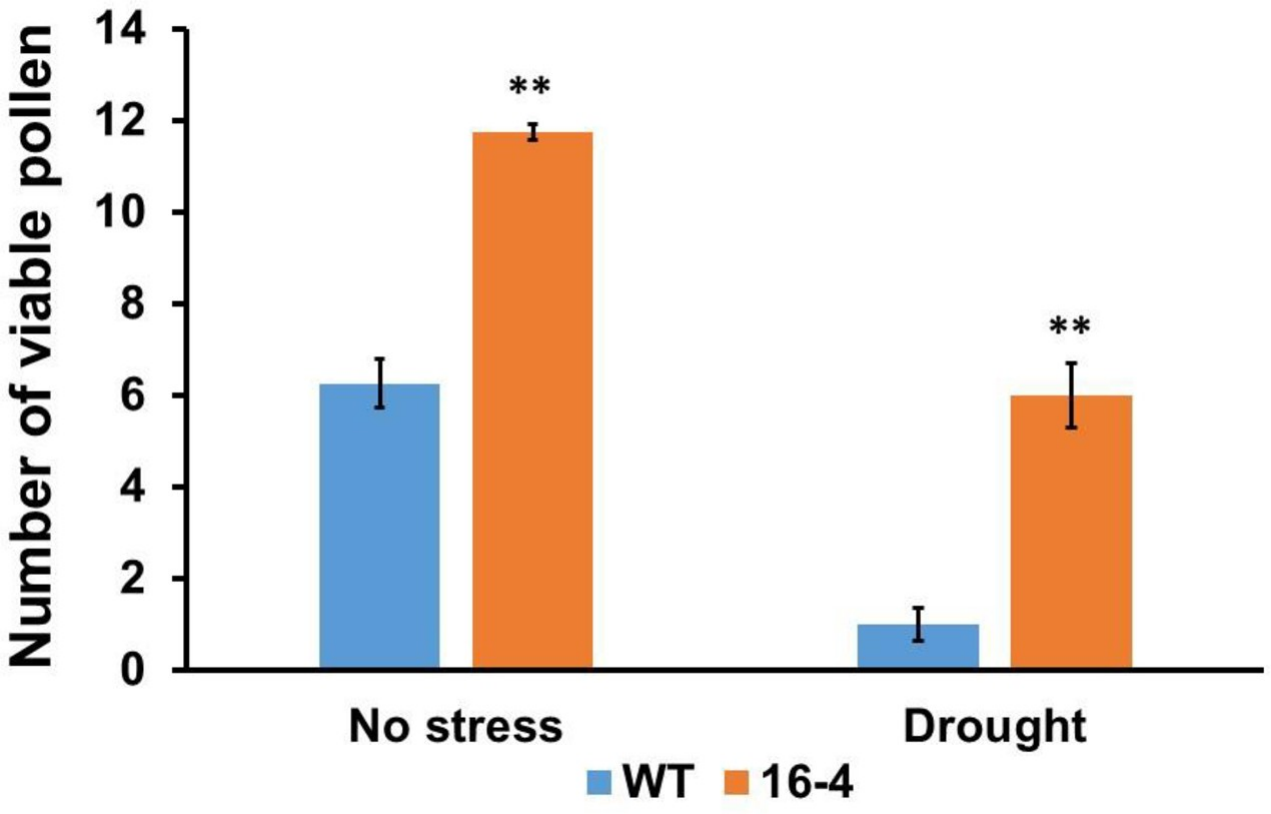
The number of viable pollen (pollen viability) of ten wild-type and transgenic rice with silenced *OsSYT-5* gene. The viable pollen ratio between transgenic and wild-type plants was measured before and after 30 days of the water deficit stress experiment. Values are mean ±SE (**P, 0.01). Data were analyzed statistically by one-way ANOVA (Analysis of Variance) with post-hoc Tukey HSD (Honestly significant difference) using SAS software.

Pollen viability was also examined under normal and drought conditions in the transgenic lines and the wild type using an Amscope microscope (Figs 10 and S4). It was noted that transgenic rice plants with the silenced *OsSYT*-5 gene had higher pollen viability compared to the wild type under both normal and drought conditions (Figs 10 and S4). The viable pollen ratio of transgenic lines to WT was found to be around 2:1 under regular watering conditions and 6:1 under drought conditions. results are valuable since higher pollen viability is another major contributor to increased crop productivity [32].

### Assessment of the impact of *OsSYT-5* gene silencing in rice on ABA signaling

**Fig 11:**
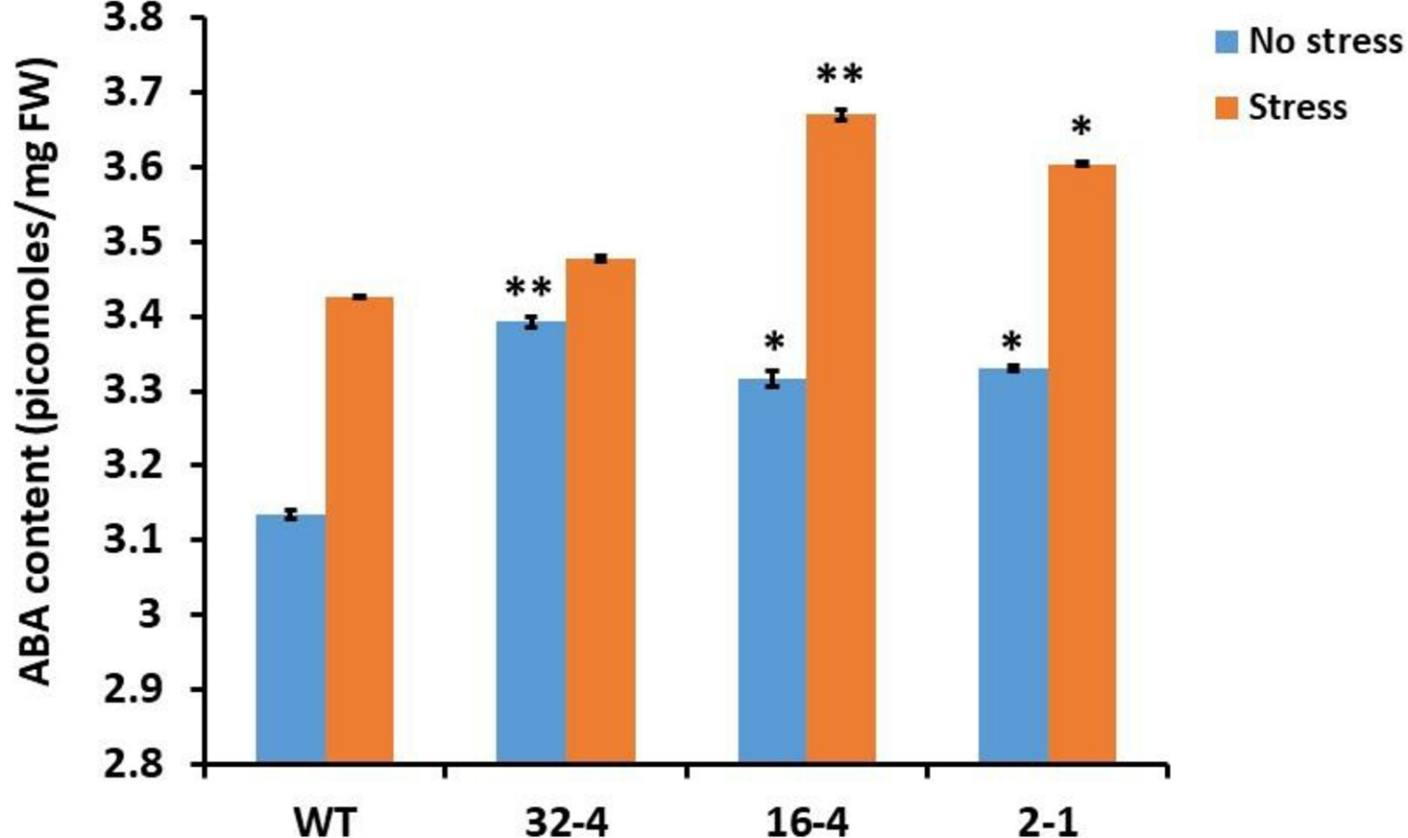
Effect of *OsSYT-5* gene silencing on ABA level in transgenic rice lines at the stage of 3-month-old young plants grown under regular watering conditions and after 30 days of water deficit stress. Values are mean ± SE (*P, 0.05, **P, 0.01). *, ** indicates a significant difference in the level of ABA compared to the wild type under both regular and drought stress conditions. Data were analyzed statistically by one-way ANOVA (Analysis of Variance) with post-hoc Tukey HSD (Honestly significant difference) using SAS software.

Abscisic acid is a phytohormone that plays an important role in plant abiotic stress response by regulating various phenotypical processes during stress [33]. To clarify the links between the ABA level and drought stress response, the ABA level in both the transgenic and wild-type plants were quantified. We found that the ABA content was elevated in the transgenic rice compared to the wild type under both regular and water deficit conditions of cultivation (Fig 11).

**Fig 12:**
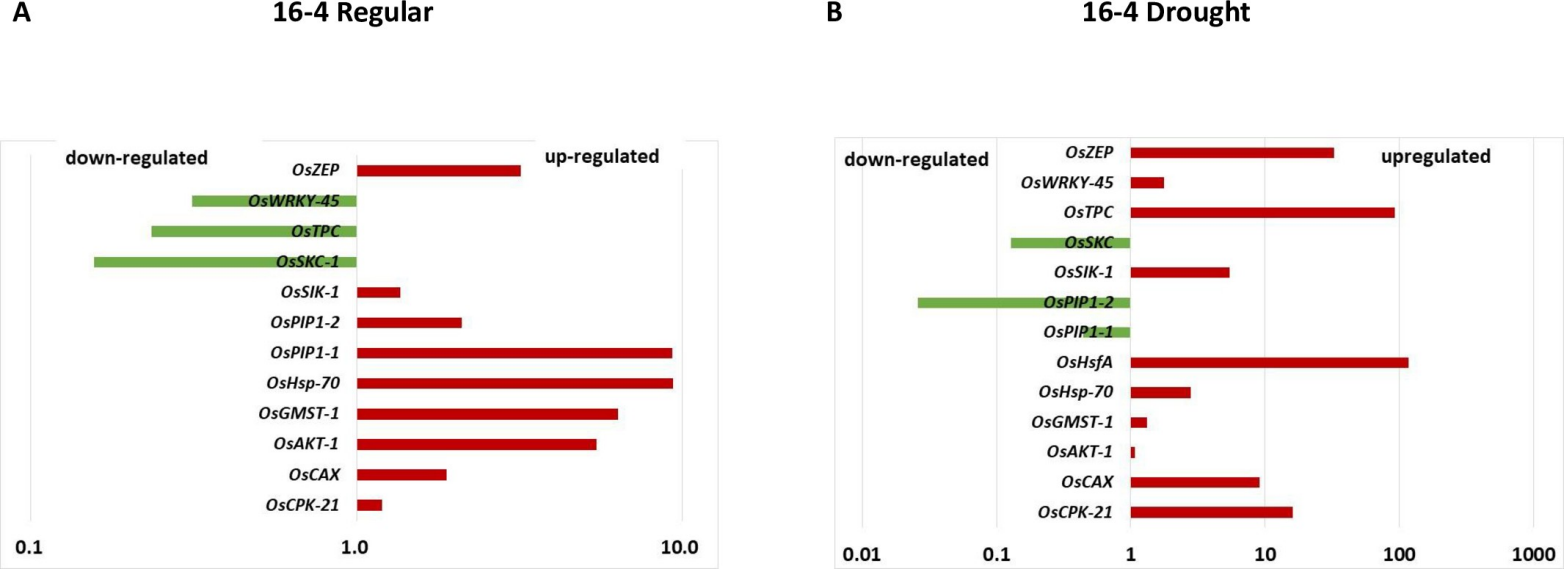
Summary of trends to up- or down-regulation of *OsZEP*, *OsWRKY-45*, *OsHsfA*, *OsSKC-1*, *OsAKT-1*, *OsCAX*, *OsTPC-1*, *OsGMST-1*, *OsPIP1-1*, *OsPIP1-2*, *OsHsp-70*, *OsSIK-1* and *OsCPK-21* genes in 16–4 transgenic line with silenced *OsSYT*-5 gene under regular (A) and water deficit conditions (B).

We can hypothesize that the *OsSYT-5* gene may play the role of a negative regulator of ABA signaling. In this case, the silencing of the *OsSYT-5* gene should affect the expression of rice genes linked to the abiotic stress response. To clarify this, the expression of 13 rice genes (*OsZEP*, *OsWRKY-45*, *OsHsfA*, *OsSKC-1*, *OsAKT-1*, *OsCAX*, *OsTPC-1*, *OsGMST-1*, *OsPIP1-1*, *OsPIP1-2*, *OsHsp-70*, *OsSIK-1*, and *OsCPK-21)* that are linked to ABA signaling according to the literature data [34–36] was monitored. Expression of selected genes in the rice wild type and transgenic lines under both normal greenhouse and drought conditions were investigated by real-time PCR. It was discovered that 12 genes of the 13 genes tested showed a trend in expression that was different from the wild type (Figs 12A and S5) under normal conditions. At the same time, all 13 genes were expressed differently in the transgenic lines compared to the wild type under drought conditions (Figs 12B and S6). The results showed that under normal conditions of plant cultivation, nine genes (*OsZEP*, *OsAKT-1*, *OsCAX*, *OsGMST-1*, *OsPIP1-1*, *OsPIP1-2*, *OsHsp-70*, *OsSIK-1*, *and OsCPK-21)* were upregulated and three genes (*OsWRKY-45*, *OsSKC-1*, *OsTPC-1)* were downregulated in the transgenic rice lines. Under drought conditions, 10 genes (*OsZEP*, *OsWRKY-45*, *OsHsfA*, *OsAKT-1*, *OsCAX*, *OsTPC-1*, *OsGMST-1*, *OsHsp-70*, *OsSIK-1*, *and OsCPK-21)* were upregulated and three genes (*OsSKC-1*, *OsPIP1-1*, *and OsPIP1-2)* were down-regulated in the transgenic lines. Data of expression analysis were summarized in Figs 12A and 12B and S7 and S8.

## Discussion

Drought is the major abiotic stress that decreases crop production around the world [37]. One of the aims of modern plant genetic engineering is the creation of stress-tolerant plants with high grain yields. This study demonstrated that the silencing of the *OsSYT-5* gene encoding synaptotagmin-5 in rice improved the drought tolerance of the transgenic plants while providing new insight for understanding the functions of synaptotagmin-5 in plants and demonstrating a potential biotechnological way to improve stress tolerance in crops. The functions of synaptotagmins are not studied well in plants. There are several reports focused on the clarification of the functions of synaptotagmin-1 in *Arabidopsis* [20, 21]. However, such reports cannot provide data on the functions of other synaptotagmins described for *Arabidopsis* or the wide range of agricultural crop species that share sequence homology between different synaptotagmins with synaptotagmins in *Arabidopsis*. For instance, synaptotagmin-1 and synaptotagmin-5 have only 30.69% sequence similarity in *Arabidopsis*. Also, the sequence similarity between studied *Arabidopsis* synaptotagmin-1 and rice synaptotagmin-5 is only 29.64%. Thus, the functions of each synaptotagmin in crops can be identified only on an individual basis using experimental approaches.

Here, the silencing of the *OsSYT-5* gene encoding rice synaptotagmin-5 resulted in a significant improvement of photosynthetic performance and drought tolerance of rice plants. The enhancement of drought tolerance of the established transgenic lines can be linked to several phenotypical traits observed in transgenic lines with the silenced *OsSYT-5* gene. Thus, the observed improved photosynthetic rate can be attributed to the increased leaf area (Fig 2A) of the transgenic plants compared to the wild type. The increased photosynthetic performance (Fig 5A) along with the significant reduction of transpiration rate (Fig 5C) in the transgenic plants might have led to enhanced rice productivity. Documented suppression of stomatal conductance and transpiration rate associated with the enhanced photosynthetic rate (Fig 5) is not the common trend for plants grown in regular conditions of cultivation. It is known that in well-watered conditions, stomatal conductance is a major determinant of the photosynthetic rate in rice [38]. However, in conditions of water deficit, the photosynthetic rate can be drastically affected by non-stomatal factors such as water availability [39]. For example, Li et al., (2017) reported about maintenance of higher ear photosynthetic rate in drought-resistant wheat through improved WUE caused by decreasing stomatal conductance and transpiration rate [39]. Significant enhancement of photosynthetic rate and WUE linked to decrease of stomatal conductance and transpiration rate was also observed in *AtEDT1/HDG11* drought-tolerant transgenic rice lines [32]. In our experiments, the observed reduction in transpiration rate is further supported by the documented decrease in leaf water loss and increased relative water content in the leaves of the transgenic lines compared to the wild type under water deficit stress conditions (Fig 6). This change can be attributed to the closing of stomata during the drought stress conditions due to the observed increase of ABA production (Fig 11), which can be responsible for the closing of stomata and the decrease of transpiration [40]. All these phenotypical changes along with the improved root morphology (deep and thicker roots improve soil water absorption) might have led to the improved drought tolerance in the *OsSYT-5* silenced transgenic lines under both normal and water-deficit stress conditions.

The silencing of the *OsSYT*-5 gene in rice also improved the panicle and tiller quality which is an important factor for enhanced overall grain productivity [41]. Apart from this, the higher viable pollen ratio in the transgenic plants compared to that of the wild-type can directly contribute to an increased seed setting rate [32]. The underlying mechanism for this increased pollen viability in transgenic rice lines awaits further investigation.

Observed changes in phenotype, photosynthetic performance, and stress tolerance of rice lines with silenced *OsSYT-5* genes are experimental evidence of the existence of molecular links between synaptotagmin-5 and ABA signaling. Taking into account that the ABA level is elevated in the transgenic rice line, we attempted to understand if the removal of expression of the *OsSYT-5* gene will affect ABA-related transcriptomes. The expression of 13 rice genes associated with ABA signaling was monitored (Figs 11 and S7 and S8). Multiple reports indicated that *OsZEP*, *OsWRKY-45*, *OsHsfA*, *OsSKC-1*, *OsAKT-1*, *OsCAX*, *OsTPC-1*, *OsGMST-1*, *OsPIP1-1*, *OsPIP1-2*, *OsHsp-70*, *OsSIK-1*, and *OsCPK-21* genes or its *Arabidopsis* homologs are directly or indirectly linked to various biotic and abiotic stress signaling pathways. For example, the upregulation of the *Oryza sativa* zeaxanthin epoxidase *(OsZEP)* gene can lead to an increase in the tolerance of rice to drought stress by enhancing the recovery of net photosynthetic rate, stomatal conductance, and transpiration rate [36]. Similarly, the over-expression of the stress-induced transcription factor, *OsWRKY-45*, enhances the drought tolerance in *Arabidopsis*, thereby indicating that this protein may be involved in the signaling pathways of the abiotic stress response [34]. Chauhan et al., (2013) have shown that *Arabidopsis* plants overexpressing the wheat heat stress transcription factor A *(HsfA*) gene, had increased tolerance to high temperature, salinity, and drought stresses [42]. Likewise, the overexpression of the *Arabidopsis* potassium transporter *(OsAKT-1)* gene reduces the sensitivity to osmotic/drought stress in transgenic plants [43]. It is also noted that the expression of the cation/H+ exchanger (*OsCAX)* gene in rice reduces the Na^**+**^ influx, thereby improving the salt tolerance in rice [44]. It was reported that the *Oryza sativa* two-pore channel *(OsTPC-1)* gene plays an important role in the regulation of cytosolic Ca^2+^ rise and innate immune responses [45]. Also, the reduced expression of the *Oryza sativa* monosaccharide transporter *(OsGMST-1)* gene confers hypersensitivity to salt stress in rice [46]. Comparably, it is also shown that the expression of *Oryza sativa* plasma membrane intrinsic proteins (*OsPIP1-1and OsPIP1-2*), increases the water deficit stress tolerance in rice [47, 48]. The *Arabidopsis* transgenic plants expressing the *T*. *harzianum* heat shock protein-70 (*Hsp-70)* gene exhibited enhanced tolerance to heat stress in previous studies [49]. Various receptor-like kinases, like stress-induced protein kinase (*OsSIK-1*), have improved the drought and salt tolerance in rice by activating the antioxidative system [50]. Previous research has suggested that *OsCPK-21 (Oryza sativa* calcium-dependent protein kinase gene) is involved in the positive regulation of the signaling pathways that are involved in the response to ABA and salt stress [35].

In this study, nine of 13 selected stress-responsive rice genes (*OsZEP*, *OsAKT-1*, *OsCAX*, *OsGMST-1*, *OsPIP1-1*, *OsPIP1-2*, *OsHsp-70*, *OsSIK-1*, *OsCPK-21)* were upregulated under normal conditions, while 10 genes (*OsZEP*, *OsWRKY-45*, *OsHsfA*, *OsAKT-1*, *OsCAX*, *OsTPC-1*, *OsGMST-1*, *OsHsp-70*, *OsSIK-1*, *OsCPK-21)* were upregulated under drought conditions and seven genes (*OsZEP*, *OsWRKY-45*, *OsSKC-1*, *OsHsp-70*, *OsCPK-21*, *OsSIK-1*, and *OsCAX)* were upregulated under both normal and drought stress conditions in the transgenic lines compared to the wild type.

It is important to note that some genes which are known to play a significant role in plant response to salt or heat stresses were upregulated under drought conditions in the studied transgenic rice lines. Multiple genomic studies revealed a considerable overlap of plant responses to osmotic stresses like drought and salinity since there is greater crosstalk between salt and drought stress signaling [51–54]. Other authors have also shown that there exist overlaps between transcripts during drought or heat stress or a combination of drought and heat stress [55–57]. Thus, the observation of the upregulation of several salt- or heat-responsive genes in rice lines with silenced *OsSYT-5* gene is not surprising.

Taking into account the enhanced level of ABA in the rice transgenic lines with silenced *OsSYT-5* gene and the results of expression analysis of genes associated with ABA-related signaling, this study confirms the existence of molecular links between ABA signaling and rice synaptotagmin 5, which is directly associated with calcium signaling through the C2 domain. Previously, the *Arabidopsis* homolog (*AtCLB)* of the *OsSYT-5* gene was described as a negative regulator of ABA signaling in *Arabidopsis* [58]. Thus, the authors hypothesize that the observed increase of drought stress tolerance in the transgenic lines with the silenced *OsSYT-5* gene can be explained by the removal of negative regulation of ABA signaling. Correspondingly, the enhanced leaf area and the increased number of stomatal files in the leaves of rice plants with silenced *OsSYT-5* gene (Fig 2), with enhanced ABA content (Fig 11) may lead to the closure of stomata and reduction of transpiration in transgenic plants during drought stress. The exact molecular mechanism of possible negative regulation of ABA signaling by synaptotagmin 5 requires further detailed investigation. However, the existence of a direct link between some C2 domain-containing proteins and ABA signaling in plants has been proven previously. For example, Rodriguez et al., (2014) showed that C2-domain CAR proteins mediate the interactions of ABA receptors with the plasma membrane and can regulate ABA sensitivity in *Arabidopsis* [59]. More clarification of the involvement of Ca^2+^ sensing proteins including those with C2-domains in the regulation of ABA signaling is needed.

## Conclusion

In this study, we have provided physiological and molecular evidence that the rice *OsSYT-5* may play a role of a negative regulator of drought tolerance in rice plants. Additionally, we have also proved that the silencing of the *OsSYT-5* gene improved yield and plant growth in the transgenic plants compared to that of the non-transgenic control plants. The regulation of various ABA-responsive genes also sheds light on the fact that the *OsSYT-5* gene may be involved in the ABA signaling pathway. Overall, we can conclude that the silencing of *the OsSYT-5* gene in rice enhanced the drought tolerance of the rice plants through the regulation of various stress-responsive genes.

## Supporting information

S1 Fig Alignment of the cloned 276 bp OsSYT-5 insert sequence with the published Os07g0409100 sequence (EMBOSS Water Pairwise Sequence Alignment) (A) and PCR confirmation of the presence of 276 bp OsSYT-5 insert in kanamycin-resistant TOP10 colonies (B). B: Lane 1- HyperLadder IV; Lane 2- Blank reaction (no template); Lanes 3-12- pENTR_OsSYT-5 colonies 1–10. 1% Agarose gel and 1X TBE stained with ethidium bromide were used for identification of insert.
(PDF)

S2 Fig Map of pANDA vector used for the creation of silencing construction containing a fragment of *OsSYT-5* gene. Vector was provided by Dr. K. Shimamoto.
(PDF)

S3 Fig Soil moisture expressed as volumetric water content (n = 10) in pots used for cultivation of wild-type and *OsSYT-5* silenced transgenic lines during 0, 5, 7, and 9 weeks of drought stress. Three-week-old young plants were used at the beginning of the drought stress experiment.
(PDF)

S4 Fig Photograph of pollen stained with iodine-potassium iodide from WT and transgenic rice line 16–4 under regular conditions (A, B) and 30 days of drought stress (C, D) taken with Amscope microscope.
(PDF)

S5 Fig qRT-PCR analysis of expression of *OsZEP*, *OsWRKY-45*, *OsHsfA*, *OsSKC-1*, *OsAKT-1*, *OsCAX*, *OsTPC-1*, *OsGMST-1*, *OsPIP11*, *OsPIP1-2*, *OsHsp-70*, *OsSIK-1 and OsCPK-21* in leaves of 6-week- old wild type and transgenic lines with suppressed *OsSYT-5* gene. Wild type and transgenic lines were grown under regular greenhouse conditions before the collection of samples. Values are mean ±SE (**P, 0.01). Data were analyzed statistically by one-way ANOVA (Analysis of Variance) with post-hoc Tukey HSD (Honestly significant difference) using SAS software.
(PDF)

S6 Fig qRT-PCR analysis of expression of *OsZEP*, *OsWRKY-45*, *OsHsfA*, *OsSKC-1*, *OsAKT-1*, *OsCAX*, *OsTPC-1*, *OsGMST-1*, *OsPIP11*, *OsPIP1-2*, *OsHsp-70*, *OsSIK-1*, *and OsCPK-21* in leaves of wild type and transgenic lines with suppressed *OsSYT-5* gene after drought stress experiment (30 days of water deficit stress). Values are mean ±SE (**P, 0.01). Data were analyzed statistically by one-way ANOVA (Analysis of Variance) with post-hoc Tukey HSD (Honestly significant difference) using SAS software.
(PDF)

S7 Fig Summary of trends to up- or down-regulation of OsZEP, OsWRKY-45, OsHsfA, OsSKC-1, OsAKT-1, OsCAX, OsTPC-1, OsGMST-1, OsPIP1-1, OsPIP1-2, OsHsp-70, OsSIK-1 and OsCPK-21 genes in 32–4 transgenic line with silenced OsSYT-5 gene under regular (A) and water deficit conditions (B).
(PDF)

S8 Fig Summary of trends to up- or down-regulation of OsZEP, OsWRKY-45, OsHsfA, OsSKC-1, OsAKT-1, OsCAX, OsTPC-1, OsGMST-1, OsPIP1-1, OsPIP1-2, OsHsp-70, OsSIK-1 and OsCPK-21 genes in 2–1 transgenic line with silenced OsSYT-5 gene under regular (A) and water deficit conditions (B).
(PDF)

S1 Raw image (PDF)

## Abbreviations

SYT: Synaptotagmin
RWC: Relative water content
WUE: Water use efficiency
VWC: Volumetric water content
ABA: Abscisic acid
SC: Stomatal conductance
PR: Photosynthetic rate
TR: Transpiration rate
qRT-PCR: quantitative RT-PCR
RT-PCR: Reverse transcription Polymerase chain reaction
AtCLB: Arabidopsis thaliana calcium-dependent lipid-binding domain
ANOVA: Analysis of variance
