# Supplementary material for: Enhancement of drought tolerance in rice by silencing of the OsSYT-5 gene
Source: PLoS One. 2021 Oct 22;16(10):e0258171. doi: 10.1371/journal.pone.0258171 (PMC8535189; doi:10.1371/journal.pone.0258171)

A

```
# Aligned_sequences: 2
# 1: EMBOSS_001
# 2: EMBOSS_001
# Matrix: EDNAFULL
# Gap_penalty: 10.0
# Extend_penalty: 0.5
#
# Length: 276
# Identity:     276/276 (100.0%)
# Similarity:   276/276 (100.0%)
# Gaps:           0/276 ( 0.0%)
# [illegible]
#
#
#=======================================

EMBOSS_001         1 caccgttggacttgtgggcactggcattgtcggcggggttggacttgttg     50
                     ||||||||||||||||||||||||||||||||||||||||||||||||||
EMBOSS_001         1 CACCGTTGGACTTGTGGGCACTGGCATTGTCGGCGGGGTTGGACTTGTTG     50

EMBOSS_001        51 gatcaggaattggtgctggtgttgggcttgttggttcgggtgttgggctt    100
                     ||||||||||||||||||||||||||||||||||||||||||||||||||
EMBOSS_001        51 GATCAGGAATTGGTGCTGGTGTTGGGCTTGTTGGTTCGGGTGTTGGGCTT    100

EMBOSS_001       101 gttggttcgggtattggcgctgtcggcagcggcctcggtaaagctgggaa    150
                     ||||||||||||||||||||||||||||||||||||||||||||||||||
EMBOSS_001       101 GTTGGTTCGGGTATTGGCGCTGTCGGCAGCGGCCTCGGTAAAGCTGGGAA    150

EMBOSS_001       151 attcatgggcaagactgtggccgggcctttcagtatgtcccggaagaacg    200
                     ||||||||||||||||||||||||||||||||||||||||||||||||||
EMBOSS_001       151 ATTCATGGGCAAGACTGTGGCCGGGCCTTTCAGTATGTCCCGGAAGAACG    200

EMBOSS_001       201 gtagcagctcaactgctccccaggctgaacaaccttctgcgtgacttgat    250
                     ||||||||||||||||||||||||||||||||||||||||||||||||||
EMBOSS_001       201 GTAGCAGCTCAACTGCTCCCCAGGCTGAACAACCTTCTGCGTGACTTGAT    250

EMBOSS_001       251 gtacagtgattgcaatggacatcgca     276
                     ||||||||||||||||||||||||||
EMBOSS_001       251 GTACAGTGATTGCAATGGACATCGCA     276
```

B

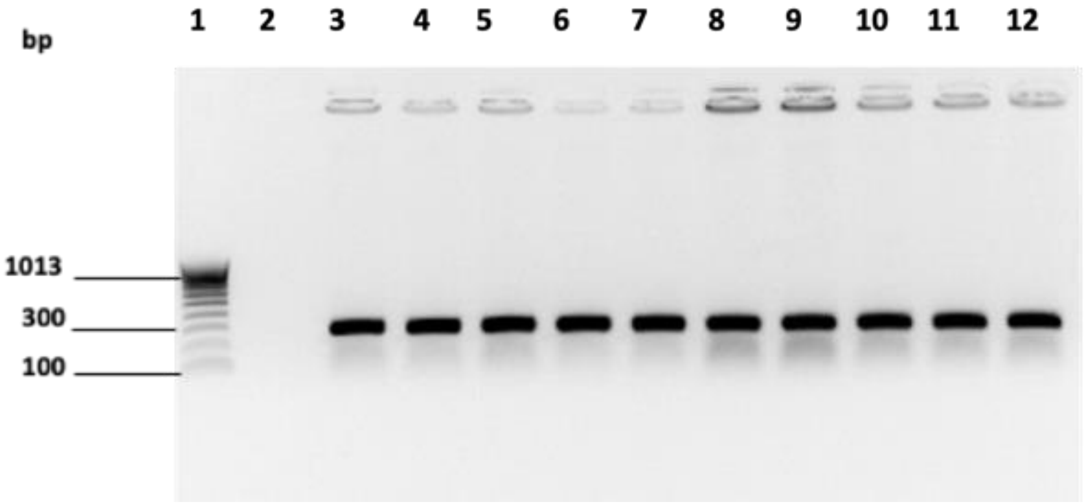

Supplement: S1 Fig — Alignment of the cloned 276 bp OsSYT-5 insert sequence with the published Os07g0409100 sequence (EMBOSS Water Pairwise Sequence Alignment) (A) and PCR confirmation of the presence of 276 bp OsSYT-5 insert in kanamycin-resistant TOP10 colonies (B). B: Lane 1- HyperLadder IV; Lane 2- Blank reaction (no template); Lanes 3-12- pENTR_OsSYT-5 colonies 1–10. 1% Agarose gel and 1X TBE stained with ethidium bromide were used for identification of insert. (PDF) [file pone.0258171.s001.pdf]
