# Supplementary figures and images for: Enhancement of drought tolerance in rice by silencing of the OsSYT-5 gene
Source: PLoS One. 2021 Oct 22;16(10):e0258171. doi: 10.1371/journal.pone.0258171 (PMC8535189; doi:10.1371/journal.pone.0258171)

# pANDA vector

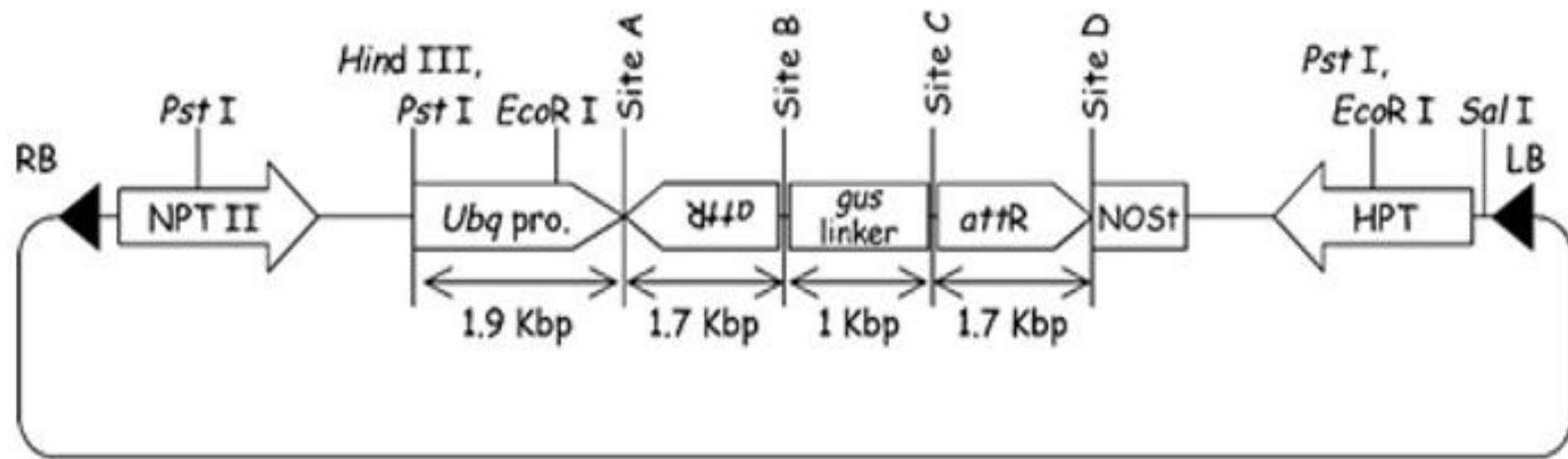

Supplement: S2 Fig — Vector was provided by Dr. K. Shimamoto. (PDF) [file pone.0258171.s002.pdf]

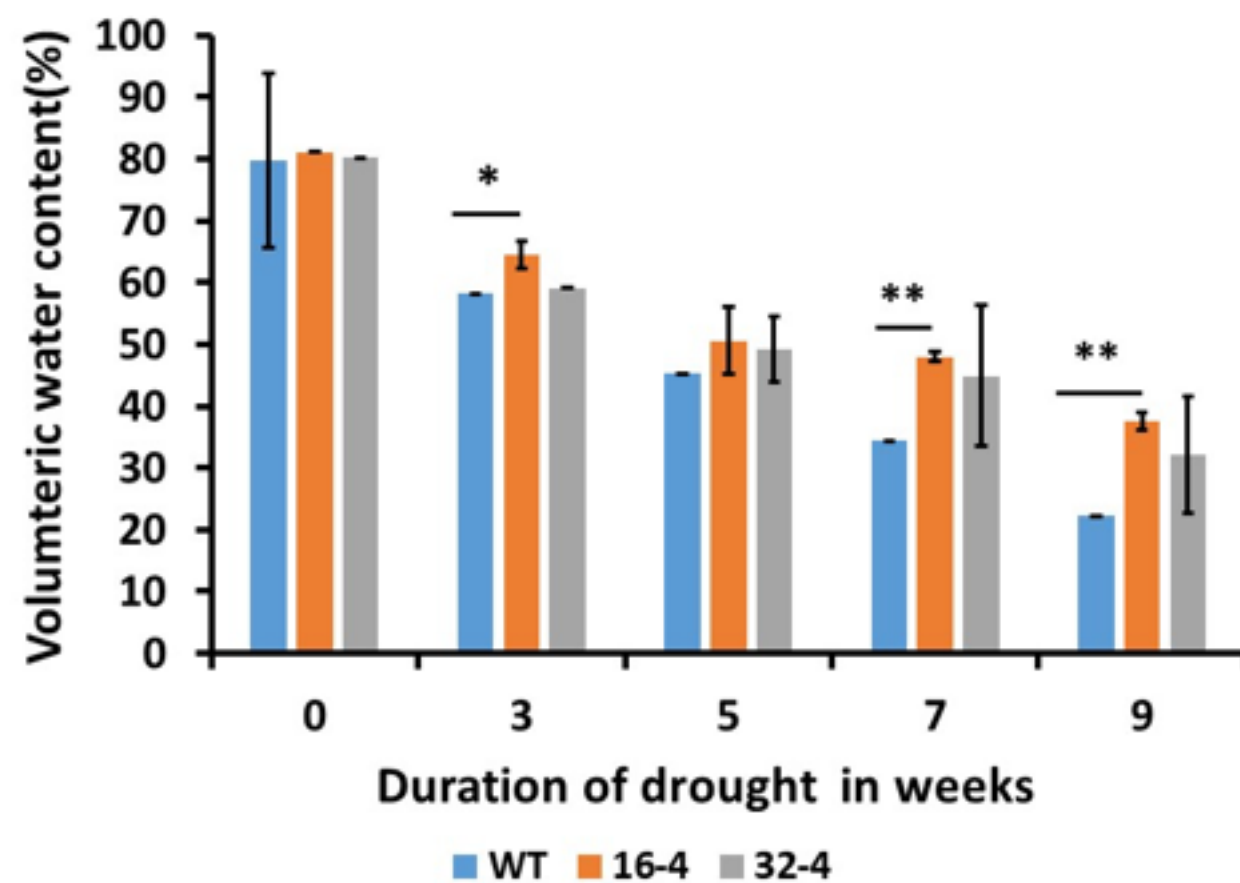

Supplement: S3 Fig — Three-week-old young plants were used at the beginning of the drought stress experiment. (PDF) [file pone.0258171.s003.pdf]

**A**

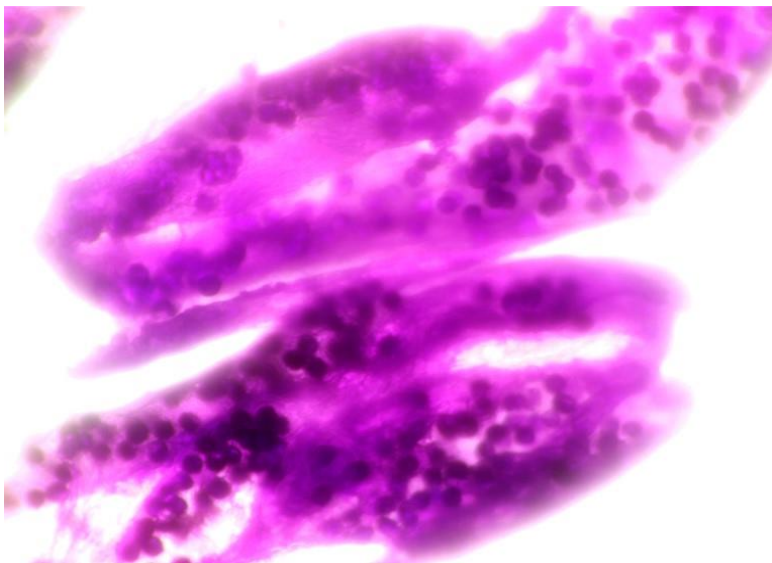

**B**

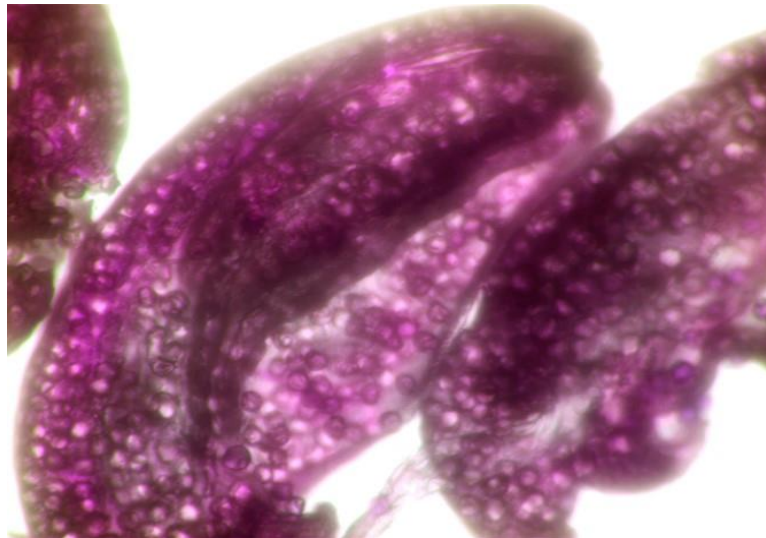

**C**

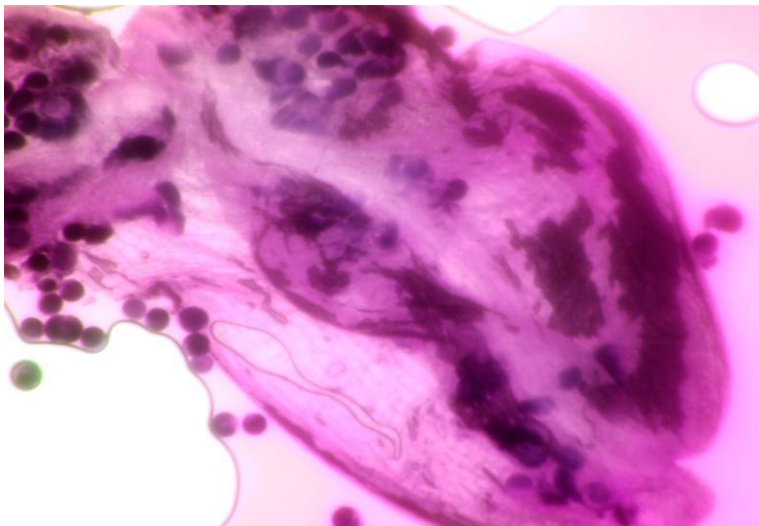

**D**

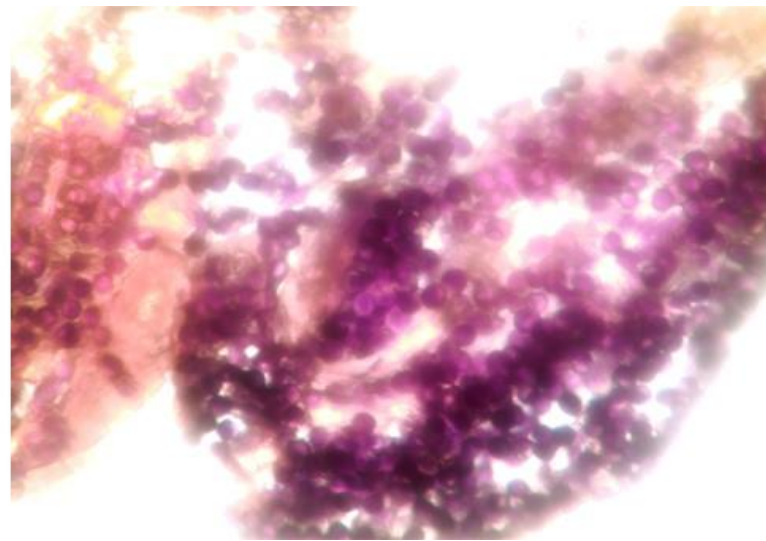

Supplement: S4 Fig — Photograph of pollen stained with iodine-potassium iodide from WT and transgenic rice line 16–4 under regular conditions (A, B) and 30 days of drought stress (C, D) taken with Amscope microscope. (PDF) [file pone.0258171.s004.pdf]

**A**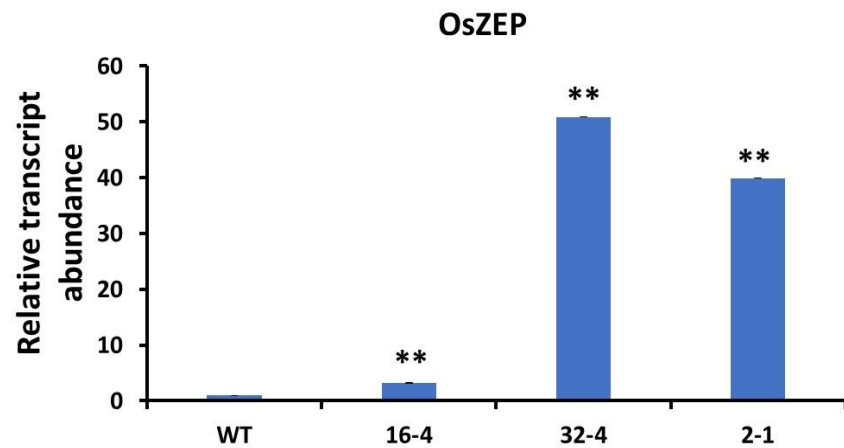**B**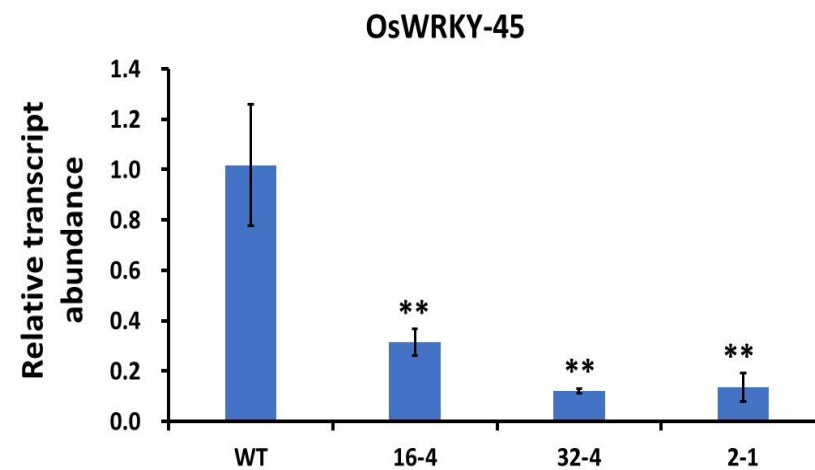**C**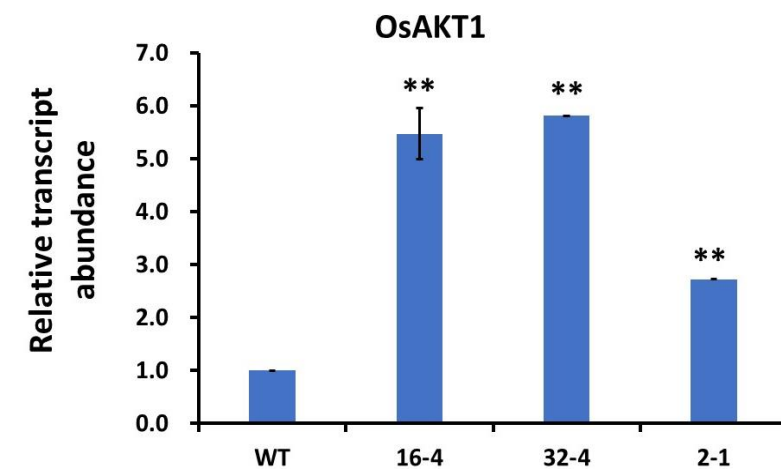**D**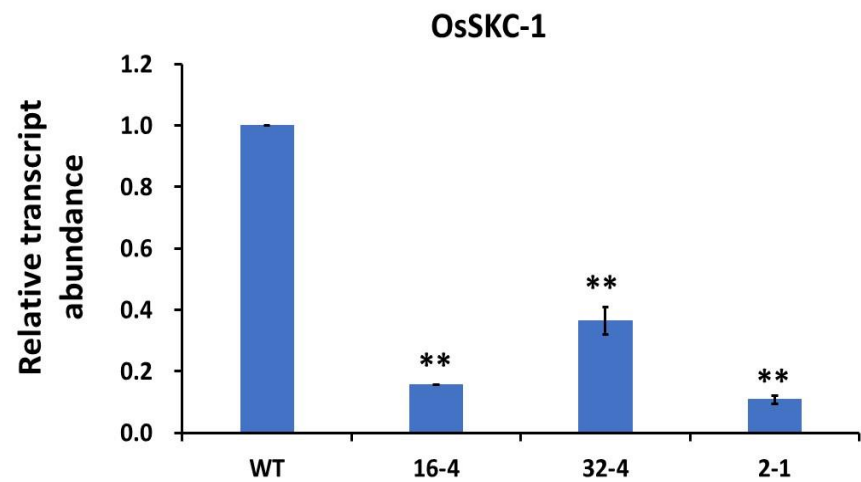**E**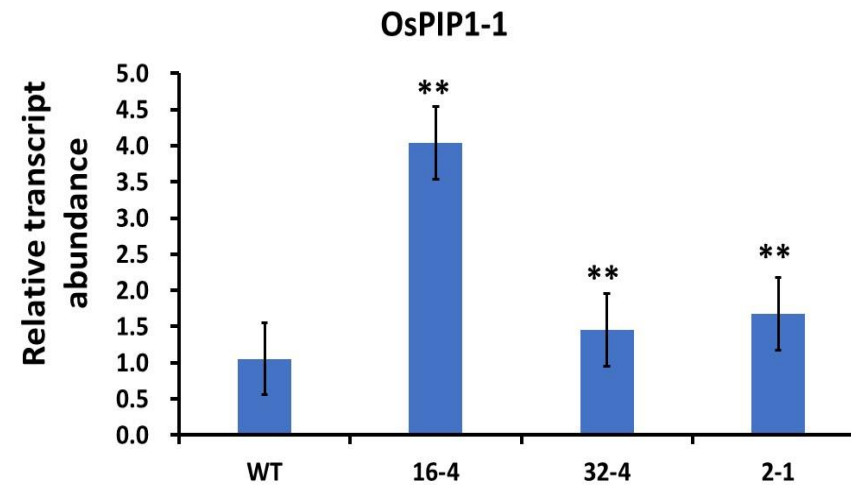**F**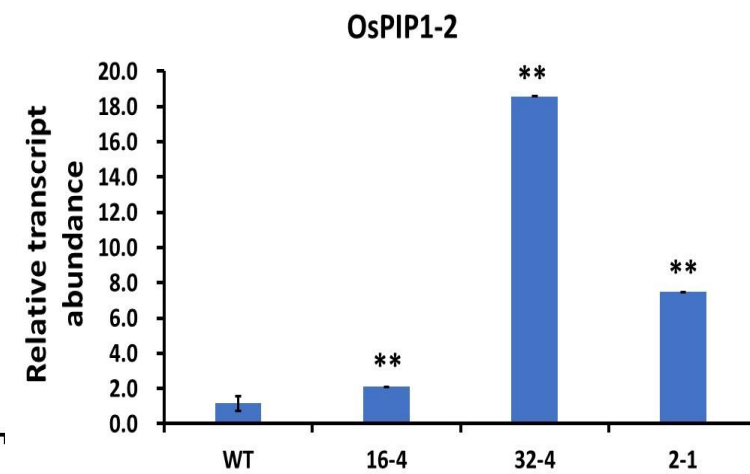

**G**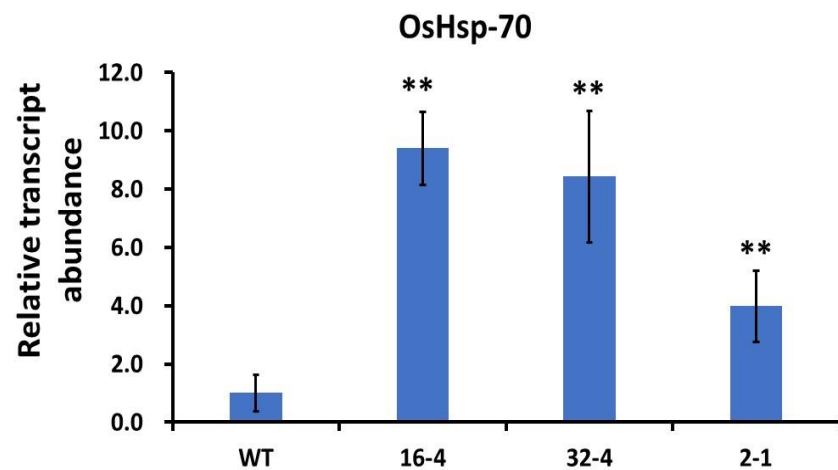**H**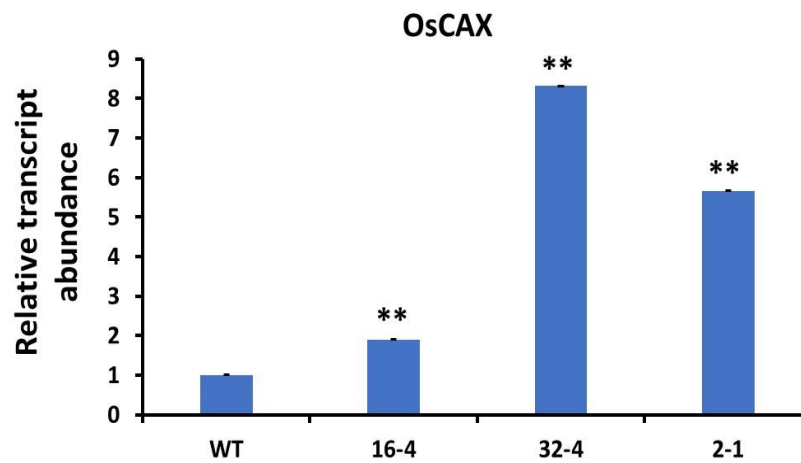**I**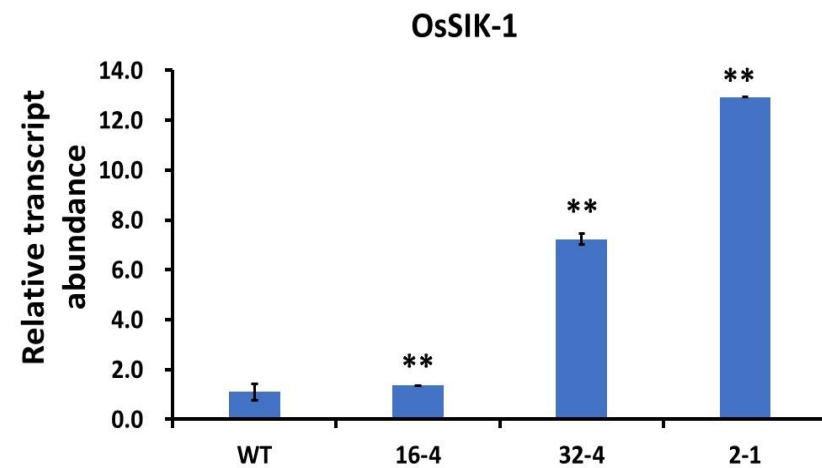**J**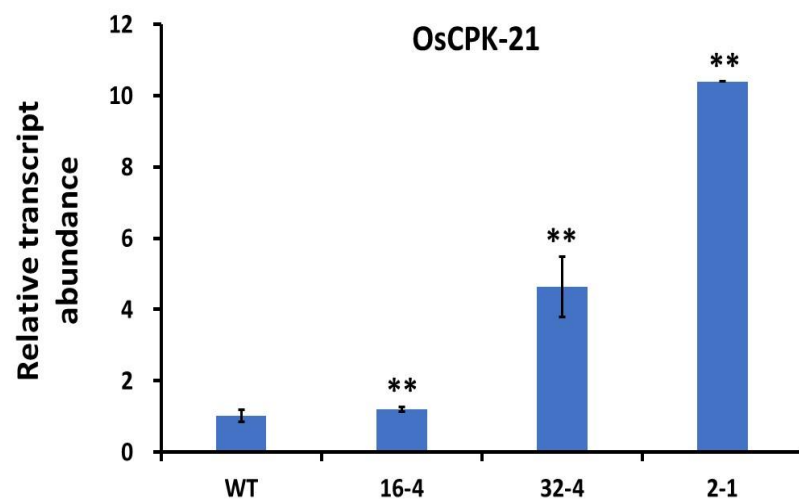**K**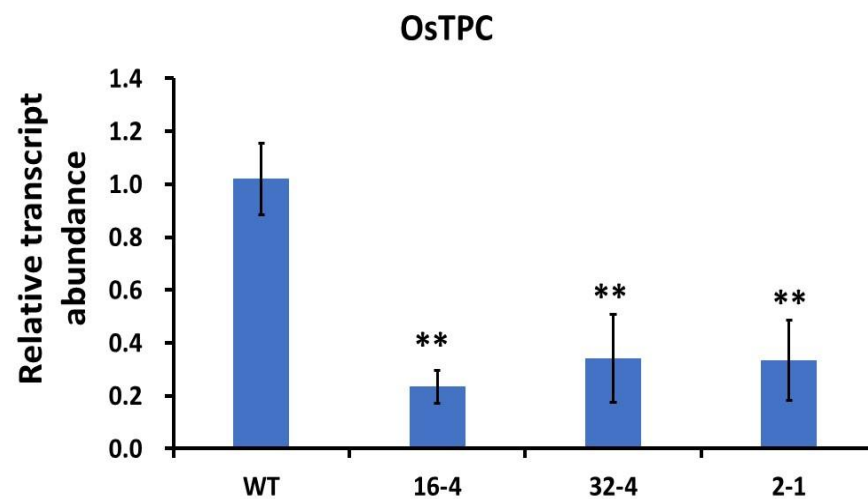**L**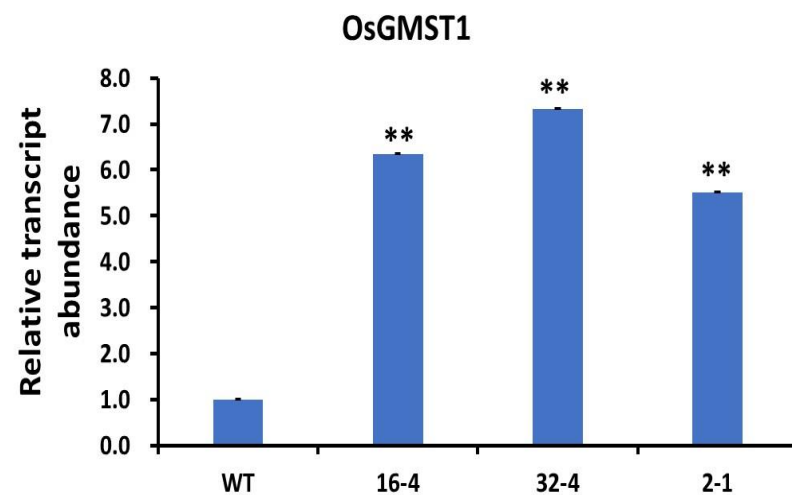

Supplement: S5 Fig — Wild type and transgenic lines were grown under regular greenhouse conditions before the collection of samples. Values are mean ±SE (**P, 0.01). Data were analyzed statistically by one-way ANOVA (Analysis of Variance) with post-hoc Tukey HSD (Honestly significant difference) using SAS software. (PDF) [file pone.0258171.s005.pdf]

**A**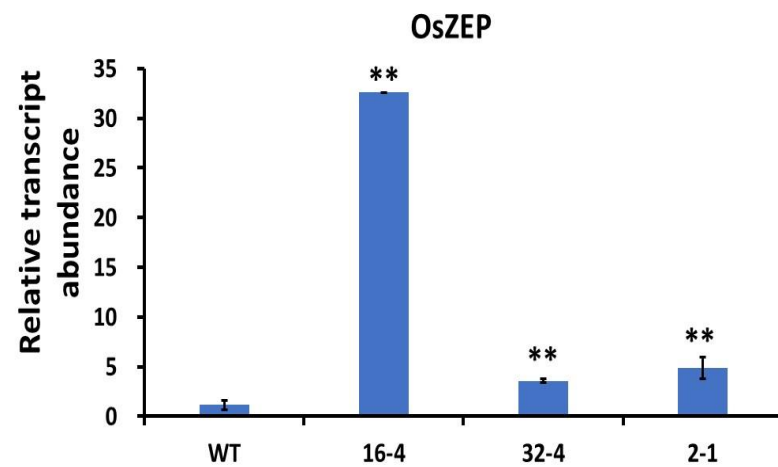**B**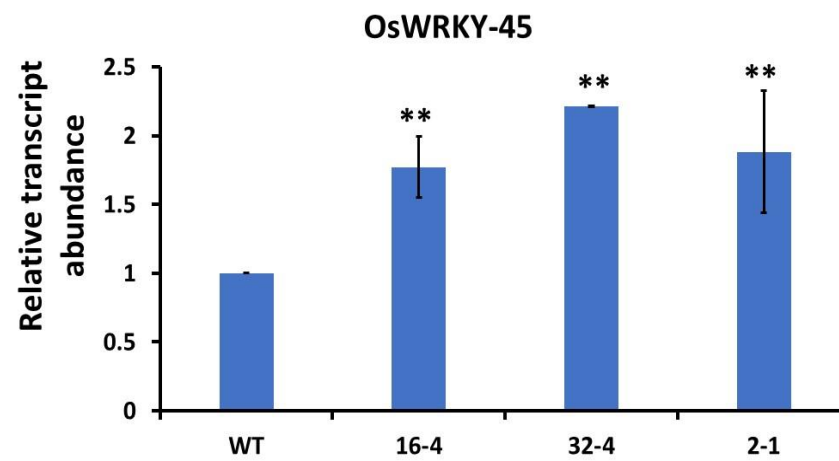**C**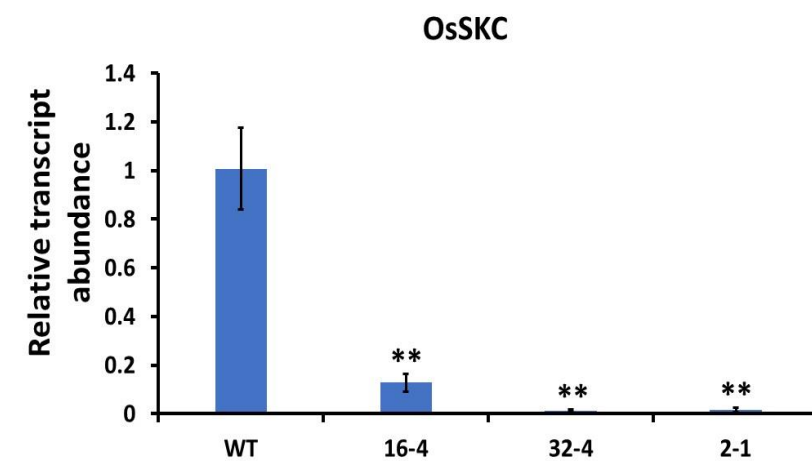**D**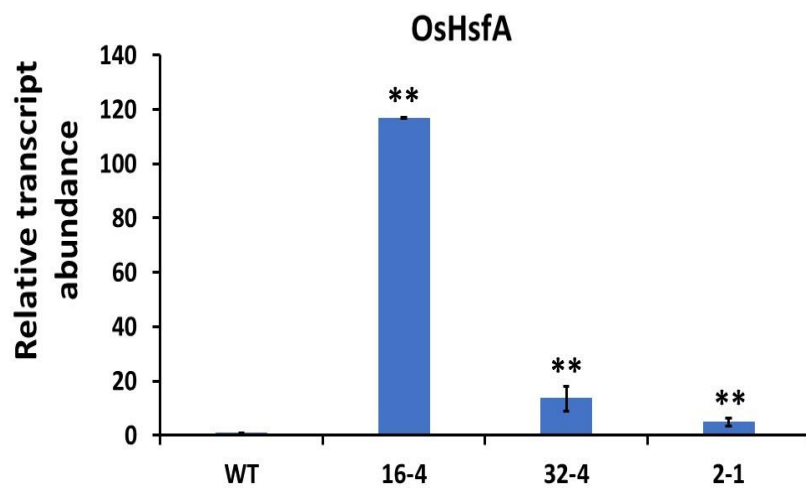**E**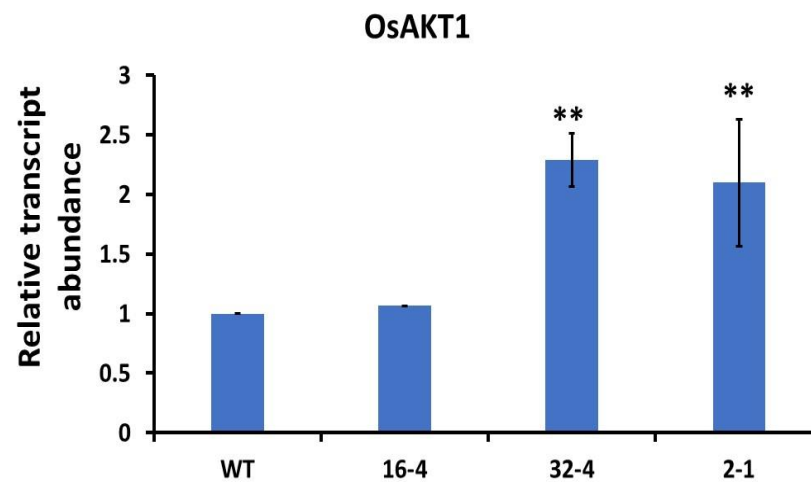**F**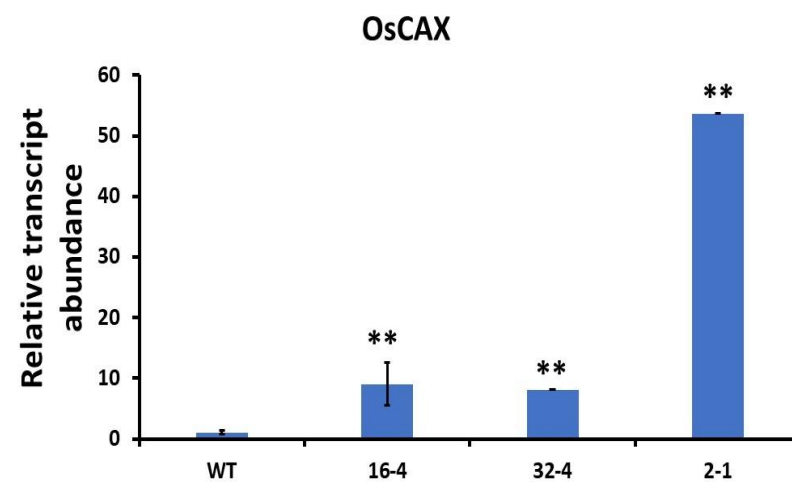

**G**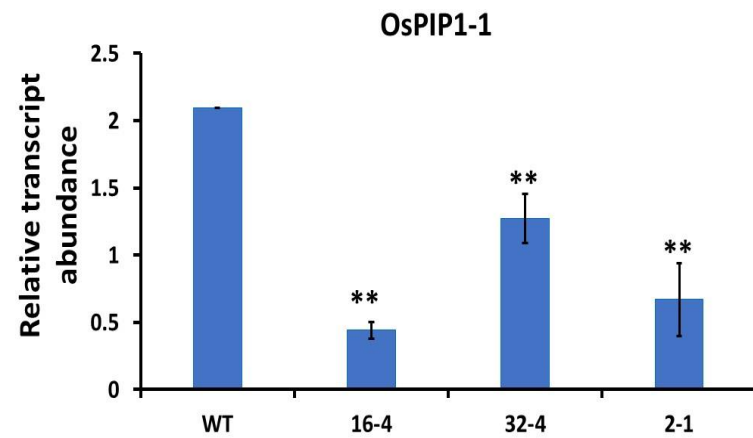**H**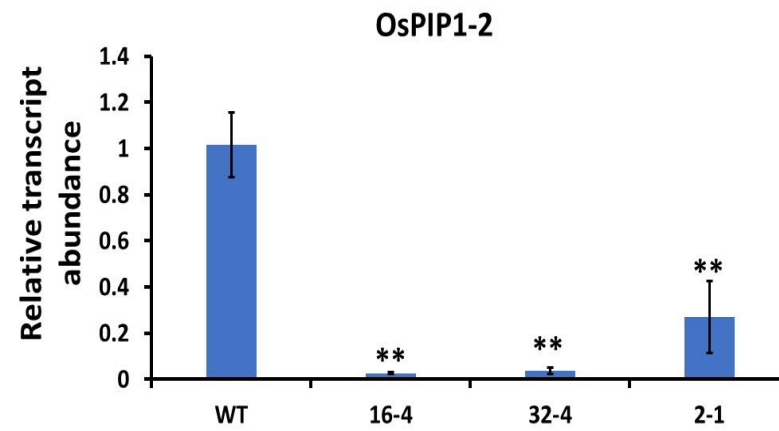**I**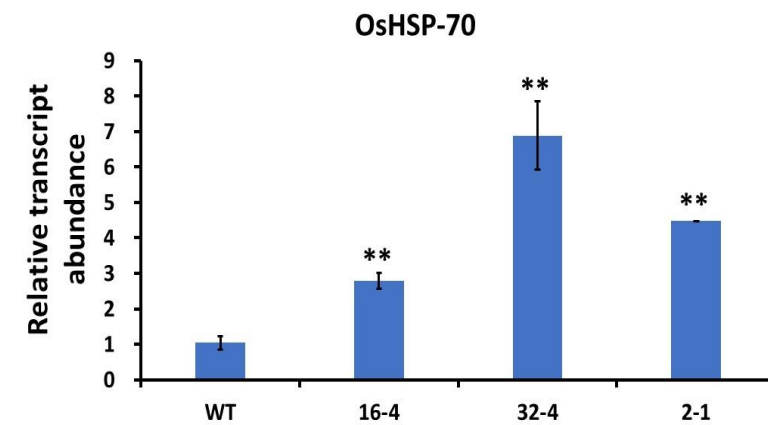**J**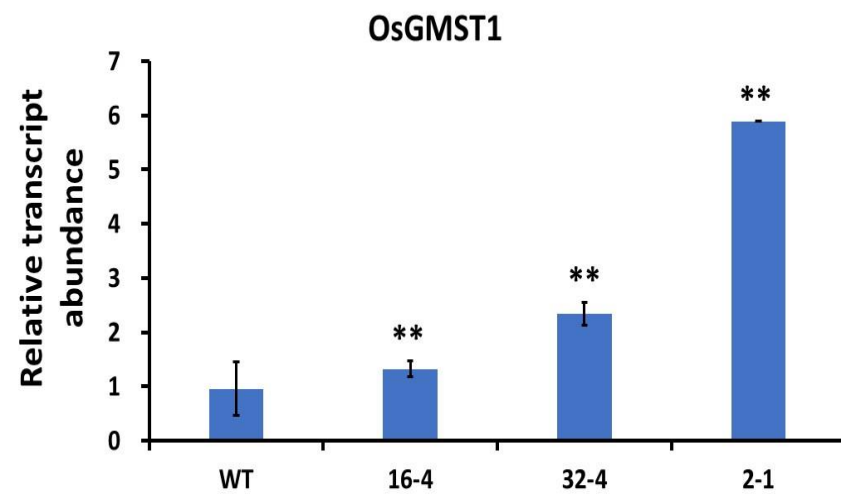**K**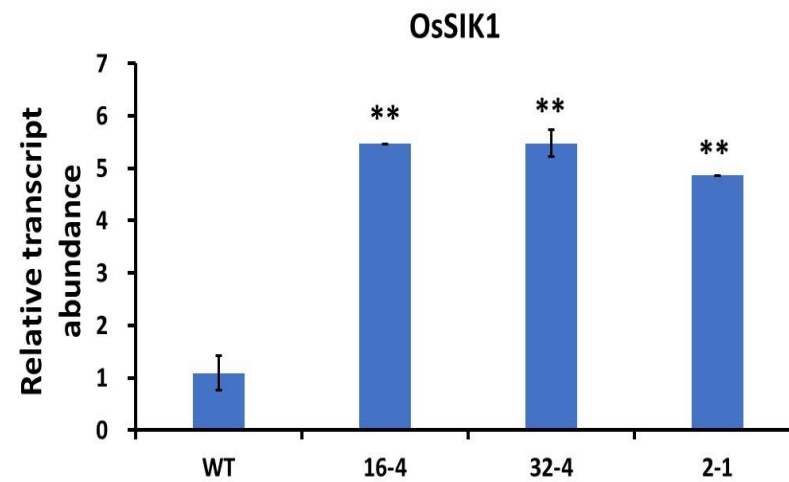**L**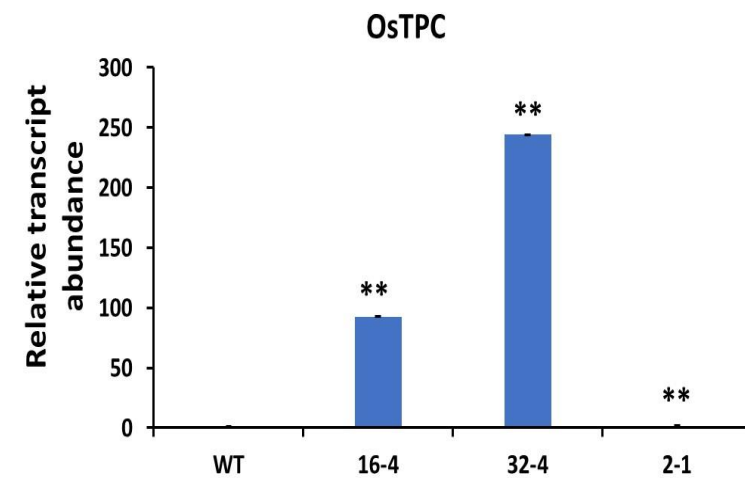

M

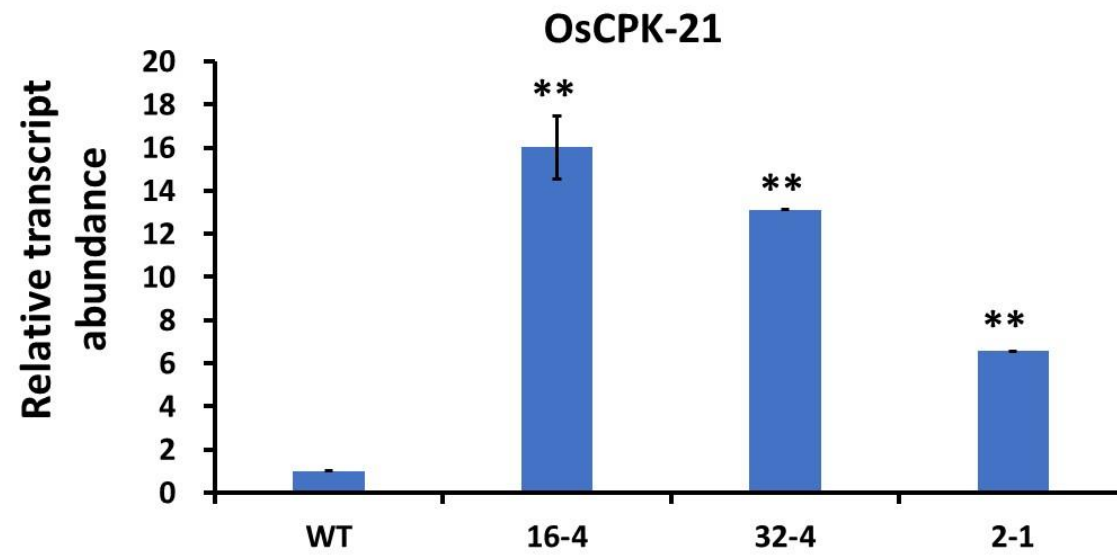

Supplement: S6 Fig — Values are mean ±SE (**P, 0.01). Data were analyzed statistically by one-way ANOVA (Analysis of Variance) with post-hoc Tukey HSD (Honestly significant difference) using SAS software. (PDF) [file pone.0258171.s006.pdf]

A

## 32-4 Regular

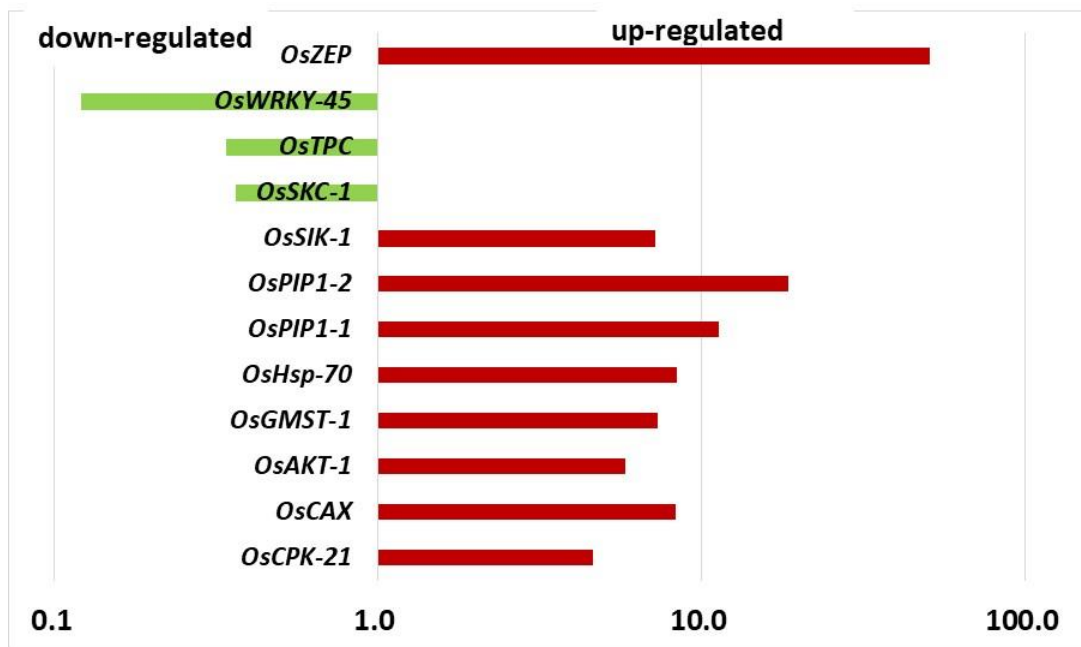

B

## 32-4 Drought

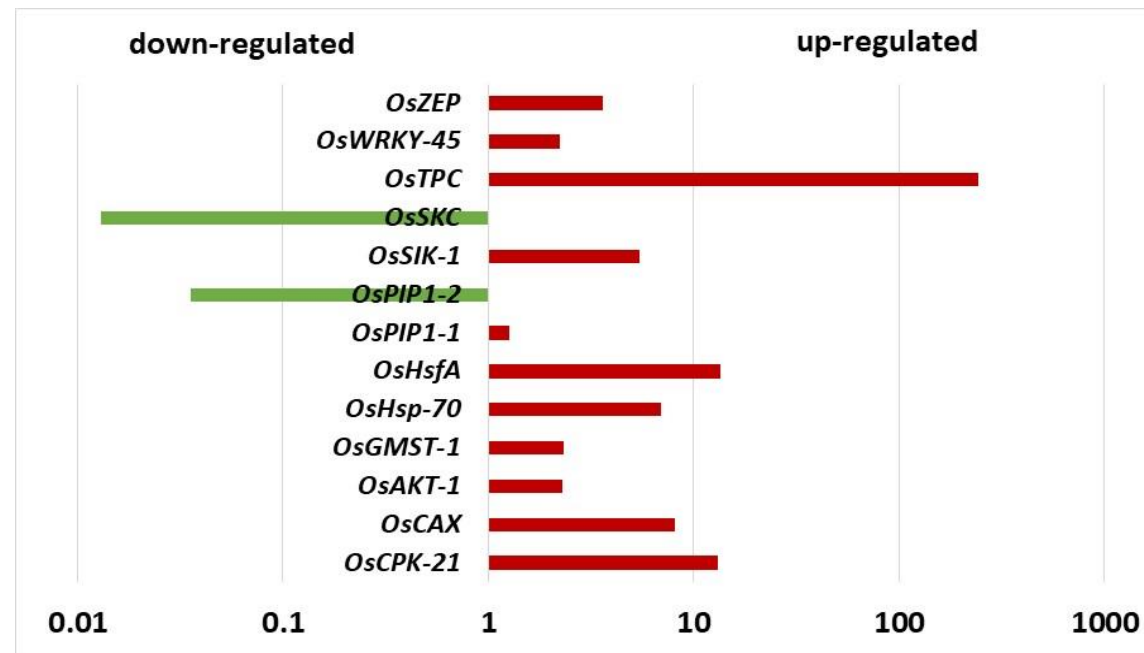

Supplement: S7 Fig — Summary of trends to up- or down-regulation of OsZEP, OsWRKY-45, OsHsfA, OsSKC-1, OsAKT-1, OsCAX, OsTPC-1, OsGMST-1, OsPIP1-1, OsPIP1-2, OsHsp-70, OsSIK-1 and OsCPK-21 genes in 32–4 transgenic line with silenced OsSYT-5 gene under regular (A) and water deficit conditions (B). (PDF) [file pone.0258171.s007.pdf]

**A**

### 2-1 Regular

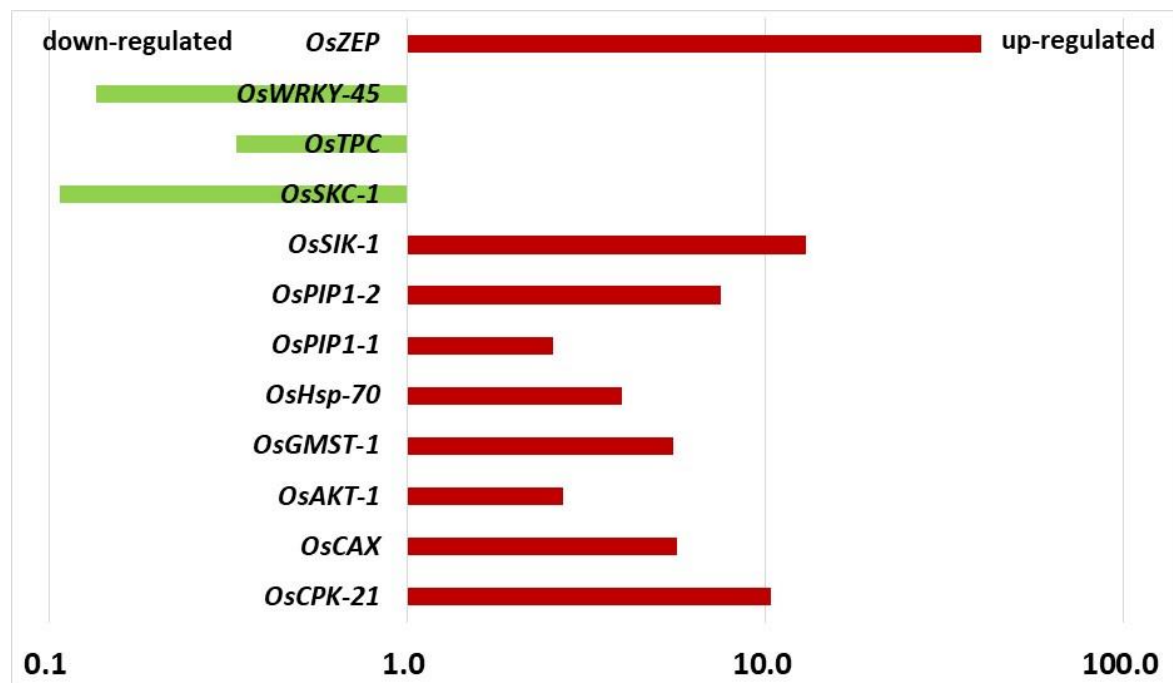

**B**

### 2-1 Drought

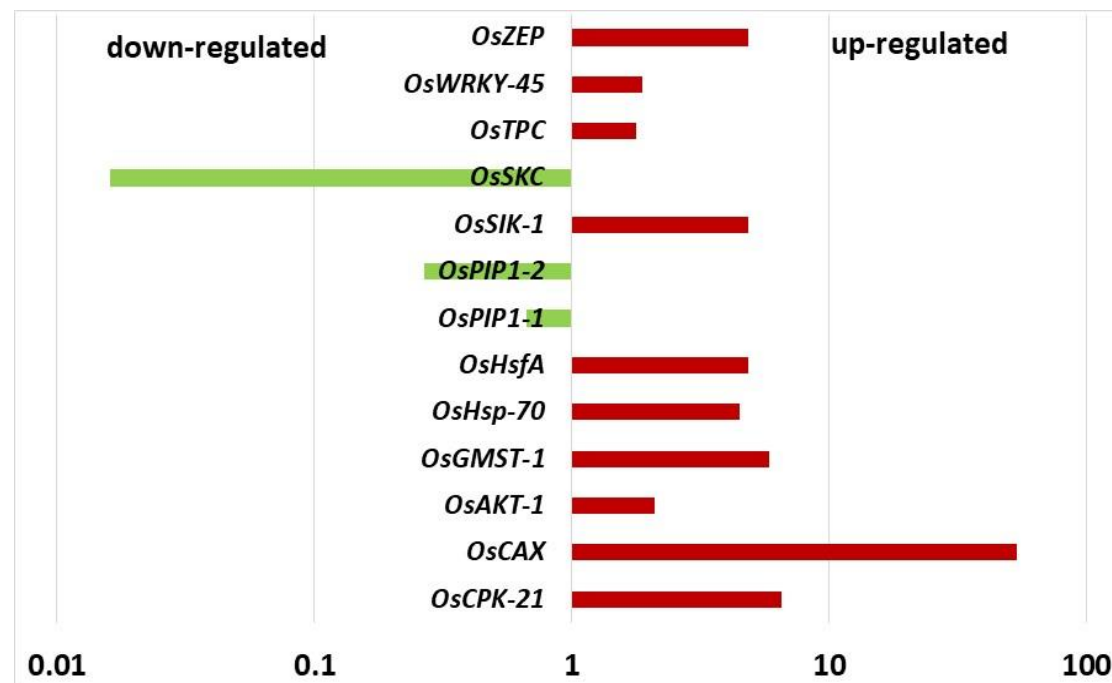

Supplement: S8 Fig — Summary of trends to up- or down-regulation of OsZEP, OsWRKY-45, OsHsfA, OsSKC-1, OsAKT-1, OsCAX, OsTPC-1, OsGMST-1, OsPIP1-1, OsPIP1-2, OsHsp-70, OsSIK-1 and OsCPK-21 genes in 2–1 transgenic line with silenced OsSYT-5 gene under regular (A) and water deficit conditions (B). (PDF) [file pone.0258171.s008.pdf]
