# Supplementary material for: Enhancement of drought tolerance in rice by silencing of the OsSYT-5 gene
Source: PLoS One. 2021 Oct 22;16(10):e0258171. doi: 10.1371/journal.pone.0258171 (PMC8535189; doi:10.1371/journal.pone.0258171)
Supplement: S1 Raw image — (PDF) [file pone.0258171.s009.pdf]

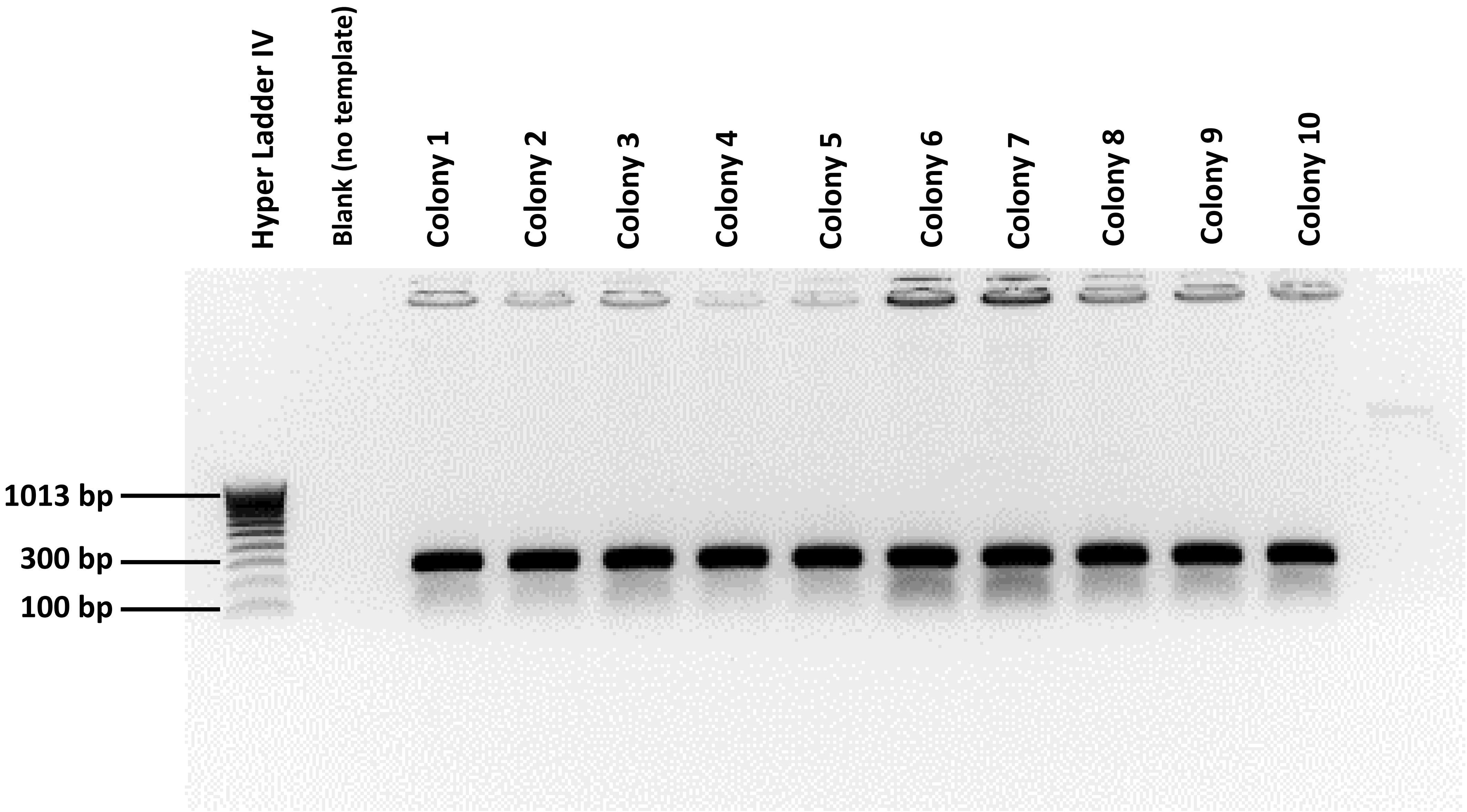

Raw image for Supporting Figure S1B. PCR confirmation of the presence of 276 bp *OsSYT-5* insert in kanamycin-resistant TOP10 colonies. The ladder size is 1Kb. 1% Agarose gel and 1X TBE stained with ethidium bromide was used. The image was captured using the Bio-Rad Gel Doc system.
